# Genetic modifiers ameliorate endocytic and neuromuscular defects in a model of spinal muscular atrophy

**DOI:** 10.1186/s12915-020-00845-w

**Published:** 2020-09-16

**Authors:** Melissa B. Walsh, Eva Janzen, Emily Wingrove, Seyyedmohsen Hosseinibarkooie, Natalia Rodriguez Muela, Lance Davidow, Maria Dimitriadi, Erika M. Norabuena, Lee L. Rubin, Brunhilde Wirth, Anne C. Hart

**Affiliations:** 1 Department of Neuroscience, Brown University, 185 Meeting Street, Mailbox GL-N, Providence, RI 02912 USA; 2 Institute of Human Genetics, Center for Molecular Medicine Cologne, Institute of Genetics, and Center for Rare Disorders, University of Cologne, Cologne, Germany; 3 Department of Stem Cell & Regenerative Biology, Harvard University, Cambridge, MA 02138 USA; 4 Department of Biological and Environmental Sciences, University of Hertfordshire, Hertfordshire, UK

**Keywords:** PLS3, hnRNP, Neurodegenerative disease, SMN, SMA, Endocytosis

## Abstract

**Background:**

Understanding the genetic modifiers of neurodegenerative diseases can provide insight into the mechanisms underlying these disorders. Here, we examine the relationship between the motor neuron disease spinal muscular atrophy (SMA), which is caused by reduced levels of the survival of motor neuron (SMN) protein, and the actin-bundling protein Plastin 3 (PLS3). Increased PLS3 levels suppress symptoms in a subset of SMA patients and ameliorate defects in SMA disease models, but the functional connection between PLS3 and SMN is poorly understood.

**Results:**

We provide immunohistochemical and biochemical evidence for large protein complexes localized in vertebrate motor neuron processes that contain PLS3, SMN, and members of the hnRNP F/H family of proteins. Using a *Caenorhabditis elegans* (*C. elegans*) SMA model, we determine that overexpression of PLS3 or loss of the *C. elegans* hnRNP F/H ortholog SYM-2 enhances endocytic function and ameliorates neuromuscular defects caused by decreased SMN-1 levels. Furthermore, either increasing PLS3 or decreasing SYM-2 levels suppresses defects in a *C. elegans* ALS model.

**Conclusions:**

We propose that hnRNP F/H act in the same protein complex as PLS3 and SMN and that the function of this complex is critical for endocytic pathways, suggesting that hnRNP F/H proteins could be potential targets for therapy development.

## Background

Neurodegenerative diseases are widespread and usually incurable. The identity of disease-causing genes is frequently known, but the mechanisms and pathways underlying disease onset, progression, and pathology remain elusive. The motor neuron disease spinal muscular atrophy (SMA) is caused by reduced levels of the survival of motor neuron (SMN) protein. Decreased SMN in SMA patients impacts the function of alpha motor neurons, resulting in their dysfunction, degeneration, and consequent muscular atrophy. SMN is required for the biogenesis of small nuclear ribonucleoproteins (snRNPs), which are critical for pre-mRNA splicing [1–3]. SMN is also found in stress granules and other RNP granules in neuronal processes [4–7], and other studies have identified roles for SMN in microRNA biogenesis and endocytic pathways [8–11]. Determining which of these various granules and RNP-related pathways are most pertinent to SMA pathogenesis is critical. To dissect this, we turn to genetic modifiers of SMA. Understanding why these modifiers suppress SMA-associated defects should reveal pathways critical for SMA pathogenesis.

Plastin 3 (PLS3) was identified as a genetic modifier in type III SMA discordant families, where six asymptomatic individuals had elevated PLS3 levels [12]. PLS3 is a calcium-dependent actin-bundling protein involved in many processes, including cell migration, adhesion, vesicle trafficking, and endocytosis [13, 14]. PLS3 overexpression ameliorates defects in many mouse, zebrafish, and tissue culture models of SMA [9, 15–18]. Because overexpression of PLS3 does not increase SMN protein levels or overtly change SMN localization, PLS3 likely acts with or downstream of SMN to suppress defects in SMA. There is no evidence that PLS3 and SMN proteins directly interact, but both pull down together in a large protein complex that also contains actin [12].

Previous work has demonstrated that decreased SMN function causes endocytic pathway defects. Endocytic defects are seen in *C. elegans*, mouse, and tissue culture models of SMA [8–10, 19]. These defects are also found in less severe models of SMA where SMN decreases are not sufficient to cause motor neuron loss. PLS3 overexpression suppresses these endocytic pathway defects in tissue culture cells and in an intermediate SMA mouse model, which was generated using antisense oligonucleotides (ASO) targeting SMN [9]. Moreover, two novel PLS3 interacting proteins have been identified and shown to be involved in the endocytosis process: overexpression of coronin 1C (CORO1C)—an F-actin binding and bundling protein—and downregulation of calcineurin-like EF-hand protein 1 (CHP1)—a calcium sensor and inhibitor of calcineurin that collectively dephosphorylates proteins involved in endocytosis—both rescue impaired endocytosis in SMA [9, 19].

Another genetic modifier, independently isolated in SMA patient families, also impacts endocytic pathway function. Neurocalcin delta (NCALD) encodes a small Ca2^+^ binding protein that acts as a neuronal calcium sensor. Loss of NCALD suppresses defects in mouse, *C. elegans*, and zebrafish SMA models, including the previously described endocytic defects [10]. Combined, the analysis of these four human genetic modifiers suggests that endocytic pathway defects in SMA may be critical contributors to SMA pathogenesis. We hypothesize that the protein complex containing SMN, PLS3, and actin may be critical for endocytic pathway function when SMN levels drop, but our poor understanding of this protein complex hinders progress.

Furthermore, understanding the interaction between PLS3 and SMN could provide insight into other neurodegenerative diseases including ataxia and amyotrophic lateral sclerosis (ALS). Indeed, recently we reported that PLS3 overexpression is beneficial in CHP1-related ataxia in mice that carry biallelic *Chp1* mutation [20]. Key proteins have been identified linking ALS and SMA. Several of these proteins function in splicing, mRNP transport, and other processes that overlap with the function of SMN [21–24]. SMN is also known to have a well-established interaction with a group of proteins known as GEMINs, and loss of GEMINs is a cellular hallmark of SMA [25]. Interestingly, studies have also shown that Gemin levels are affected in various models of ALS including SOD1 models [26–28]. These studies along with a recently published work that undertook a gene ontology enrichment analysis of published ALS modifier genes found an overlap in shared pathways that may underlie ALS and are also involved in SMA [29]. With evidence that similar pathways and genes are affected across multiple neurodegenerative diseases, it is crucial that one takes into consideration the possibility that a genetic modifier of SMA might also modify ALS.

Therefore, in this study, we focus on understanding the genetic interaction between PLS3 and SMN and testing if similar genetic interactions occur in a *C. elegans* model of ALS through behavioral analysis. We demonstrate that increased PLS3 expression suppresses locomotion defects in an established *C. elegans* model of SMA*.* Through a combination of approaches in *C. elegans* and vertebrate systems, we identify novel proteins in the PLS3/SMN complex*.* Using genetic and behavioral tools available in *C. elegans*, we demonstrate that perturbing the function of these novel proteins modifies defects in SMA models and we identify the hnRNP F/H ortholog, *sym-2*, as a novel genetic suppressor of *smn-1* loss of function defects. We find that PLS3, SMN, and hnRNP F/H are in a large protein complex in vertebrate motor neuron processes. Furthermore, we find that similar to PLS3 overexpression, decreasing hnRNP F/H suppresses defects in endocytic pathways caused by reduced SMN levels. Finally, increased expression of PLS3 or decreased expression of hnRNP F/H orthologs suppresses locomotion defects in a *C. elegans* ALS model [30]. Taken together, these results demonstrate that PLS3, SMN, and hnRNP F/H are functional members of a protein complex that is pertinent to SMA and that the mechanism underlying PLS3 or hnRNP F/H suppression is likely altering endocytic pathway activity. In addition, the suppression of ALS behavioral defects in a *C. elegans* model by increasing PLS3 or decreasing SYM-2 expression shows that conserved pathways are affected across motor neuron diseases.

## Results

### SMN function is essential for proper recovery after exhaustion

**Fig. 1:**
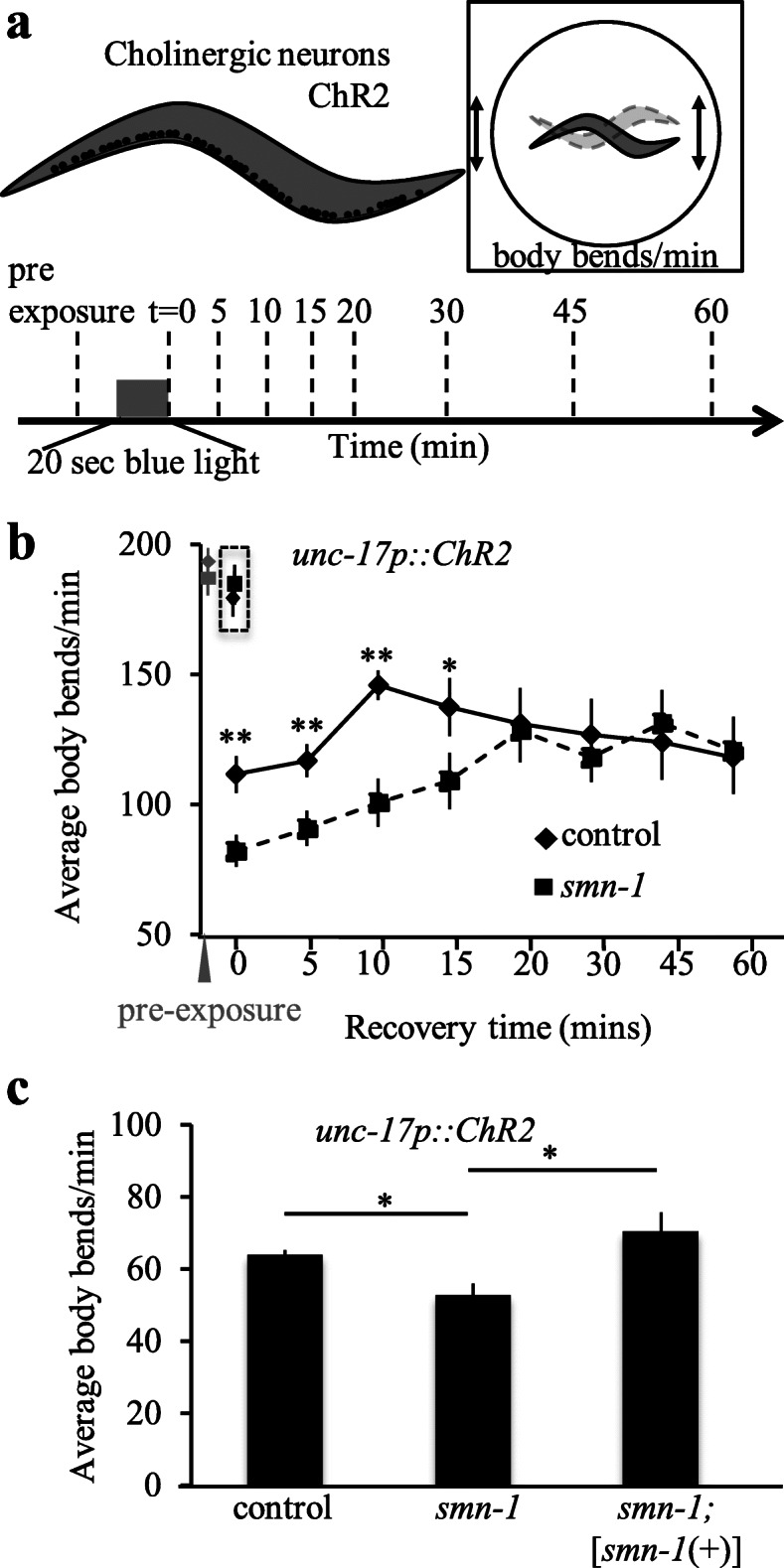
Rescue of *smn-1*(*cb131*) exhaustion defects by reintroduction of *smn-1*(+). **a** Schematic representation of experimental design. Channelrhodopsin is expressed in cholinergic motor neurons. After overnight retinal feeding, animals are exposed to blue light stimuli for 20 s in liquid and body bends per minute are recorded over time. A body bend is defined as a muscle contraction resulting in a dorsal to ventral movement across the midline of the animal. **b** Before blue light stimulation, animals have almost identical locomotion (data points shown in gray). After 20 s blue light stimulation of channelrhodopsin (ChR2) in cholinergic neurons, *smn-1*(*cb131*) partial loss of function animals had decreased locomotion rates when fed retinal and had delayed recovery to original activity. Animals not fed retinal are shown immediately following blue light exposure in a hashed box. Locomotion reported as body bends per minute in liquid. **c** Introduction of an *smn-1* genomic transgene restored normal locomotion rates immediately following blue light illumination. *[smn-1*(+)*]* is *rtSi10*, a homozygous single-copy insertion on chromosome IV (see Additional file 1: Table for details); ANOVA *F*(3.32, 12.5) = 7.27, *p* < 0.05. All animals carry *oxIs364[unc-17p::ChR2]* and exhibit no change in locomotion without exogenous retinal feeding (data not shown). The drop in body bends per minute from panel **b** to **c** is due to methodology. Panel **b** is counted manually by eye whereas panel **c** and future locomotion rates are calculated using the NABAS program described in the “Methods” section. For all locomotion assays, *n* ≥ 30 animals were used per determination and combined from 3 independent and blinded trials. Student’s *t* test: *p < 0.05, **p < 0.01. SEM indicated

The *C. elegans* genome contains a single ortholog of SMN, encoded by the *smn-1* gene*.* The severe loss of function allele, *smn-1*(*ok355*), has impaired neuromuscular function, and animals usually die during larval development, despite the fact that premature motor neuron death is not observed [31, 32]. A less severe allele, *smn-1*(*cb131*), is viable, fertile, and has only subtle defects. *smn-1*(*cb131*) is a D27N amino acid substitution, analogous to a D44V allele found in a type III SMA patient [33, 34]. The locomotion of *smn-1*(*cb131*) animals is normal on solid surfaces [35], and we observed no locomotion defects in animals swimming in liquid (assessed as body bends per minute in developmental stage matched animals, Fig. 1b pre-exposure time point, *p* = 0.746). However, *smn-1*(*cb131*) animals are resistant to immobilization by pyridostigmine bromide, an acetylcholinesterase inhibitor, suggesting latent defects in cholinergic neuromuscular junction (NMJ) synapse function [35].

Liewald and colleagues showed that channelrhodopsin (ChR2) stimulation of *C. elegans* cholinergic motor neurons depletes synaptic vesicle pools and results in transiently decreased locomotion post-stimulation [36, 37]. Using this paradigm (Fig. 1a and detailed description in the “Methods” section), we challenged NMJ function in *smn-1*(*cb131*) animals. As a result, cholinergic motor neuron stimulation in *smn-1*(*cb131*) animals revealed a locomotion defect during recovery after exhaustion, only in the presence of retinal. Before the blue light activation of cholinergic ChR2, locomotion rates of *smn-1*(*cb131*) and control animals were indistinguishable. After ChR2 activation for 20 s, locomotion rates decreased in both *smn-1*(*cb131*) and control animals. However, the post-exhaustion decrease was more profound in *smn-1*(*cb131*) animals (locomotion rates were 58% of controls, Fig. 1b, *p* = 0.001). *smn-1*(*cb131*) locomotion was restored to control levels 20 min after stimulation (Fig. 1b, *p* = 0.869), arguing against neuron or synapse damage caused by ChR2 activation and more likely a delayed recovery after exhaustion in these animals. Thus, we conclude that a latent defect in recovery after exhaustion is revealed in *smn-1*(*cb131*) by ChR2 stimulation of cholinergic neuron activity.

To test if *smn-1*(*cb131*) defects are caused by diminished SMN-1 levels, we undertook transgenic rescue studies. A single copy of the *C. elegans smn-1* gene, including promoter, exons, introns, and 3′ untranslated sequences, was inserted into another chromosome by site-directed homologous recombination, creating *rtSi10[smn-1p::smn-1]* [8]. This transgene restored normal recovery rates after exhaustion in *smn-1*(*cb131*)*;ChR2* animals (*smn-1*(*cb131*)*;rtSi10* versus *smn-1*(*cb131*), Fig. 1c, *p* = 0.043). Therefore, diminished levels of SMN-1 impair recovery after exhaustion in *smn-1*(*cb131*) animals. Next, we used this ChR2 exhaustion assay to assess the impact of PLS3, a previously identified genetic modifier [12, 16]*.*

### Increased expression of plastin in neurons suppresses *smn-1*(*cb131*) locomotion defects

PLS3 is a protective modifier of SMA; increased PLS3 levels suppress defects in humans and SMA models in zebrafish, flies, and mice [9, 12, 16, 38–40]. We determined whether increasing PLS3 levels would ameliorate various functional defects in *C. elegans* SMA models.

**Fig. 2:**
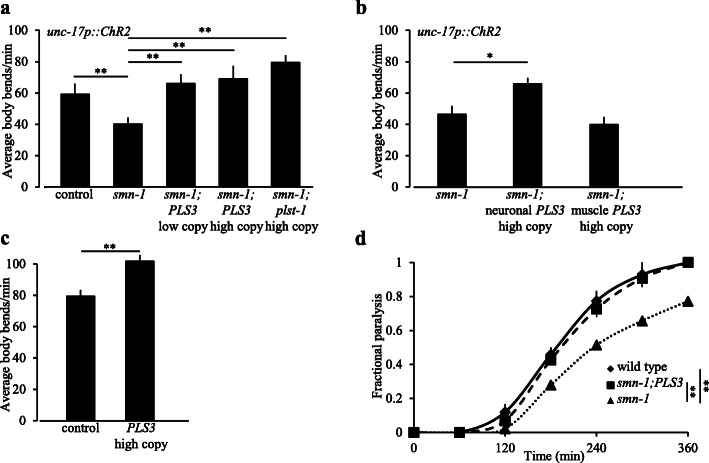
Neuronal expression of human PLS3 restores normal locomotion in *smn-1*(*cb131*) animals. **a** Locomotion rates of *smn-1*(*cb131*) animals decreased after a 20-s blue light stimulation of ChR2 in cholinergic neurons, in the presence of retinal. Introduction of somatic expression of human Plastin 3 (PLS3) restored normal locomotion rates immediately post-exhaustion. Two different PLS3 transgenes were used. Low copy is a homozygous single-copy insertion of *[dpy-30p::PLS3]*, *rtSi27* on chromosome II; high copy is an integrated multi-copy transgene incorporating the same transgene as low copy, *rtIs59.* Ubiquitous overexpression of the *C. elegans* ortholog of PLS3 (*plst-1*) was equally effective *rtEx850*. ANOVA *F*(2.9, 13.5) = 3.33, *p* < 0.05. **b** Neuronal expression of PLS3 *rtEx852 [unc-119p::PLS3]* rescued the post-exhaustion locomotion defects of *smn-1*(*cb131*) animals; expression in muscles *rtEx851 [myo-3p::PLS3]* did not. ANOVA *F*(1.56, 10.28) = 4.25, *p* < 0.05. **c** Overexpression of human PLS3 at high copy levels using *rtIs59* increased basal locomotion rates in a wild-type background compared to controls. **d** *smn-1*(*cb131*) mutant animals were resistant to the cholinesterase inhibitor aldicarb, based on slower time to paralysis. Introduction of ubiquitously expressed human PLS3 restored sensitivity to normal levels. Note that *C. elegans plst-1* was intact in all genotypes reported here and control animals contained the transgene *rtSi28 [dpy-30p::empty]* to account for insertion site and/or multi-copy promoter effects. In all assays, *n* ≥ 30 animals per determination, combined from 3 independent trials. Scorers were blinded to the genotypes of animals during the collection and analysis of data for the locomotion and aldicarb sensitivity assays. Student’s *t* test, Mann-Whitney *U* test (**a**–**c**), or log rank test (**d**): *p < 0.05, **p < 0.01. SEM indicated

First, we assessed locomotion after ChR2 exhaustion to determine if the introduction of human PLS3 could restore locomotion when SMN-1 function is decreased. We introduced two transgenes; a single-copy transgene (*rtSi27[dpy-30p::PLS3]*) or a multi-copy transgene (*rtIs59 [dpy-30p::PLS3]*) was crossed onto the *smn-1*(*cb131*)*;ChR2* background. In both cases, human PLS3 was expressed ubiquitously in somatic tissues using the *dpy-30* promoter [41]. We found that PLS3 overexpression restored post-exhaustion locomotion rates to normal levels in *smn-1*(*cb131*) animals (after cholinergic ChR2 neuron stimulation, Fig. 2a, *p* = 0.002 and 0.003). Therefore, we conclude that expression of human PLS3 ameliorates the functional defects caused by decreased SMN-1 in this exhaustion paradigm.

To test for cross-species conservation of plastin function, we also examined the impact of overexpressing the *C. elegans* PLS3 ortholog, *plst-1*. Loss of *plst-1* exacerbates *smn-1* neuromuscular defects in other paradigms [39]. We assessed locomotion in *smn-1* mutant animals that ubiquitously express *C. elegans plst-1* mRNA at increased levels (*rtEx850 [dpy-30p::plst-1]*). The multi-copy *plst-1* transgene restored normal locomotion rates after exhaustion in *smn-1*(*cb131*)*;ChR2* animals (Fig. 2a, *p* = 0.006). Combined with previous loss of function studies [39], our results are consistent with the hypothesis that Plastin 3 and its *C. elegans* ortholog are cross-species modifiers of locomotion defects in SMA models.

SMN is broadly expressed, but its function is required in motor neurons for normal NMJ activity, based on work in mammalian [42] and *C. elegans* SMA models [31]. Plastin 3 is also broadly expressed in neuronal and non-neuronal tissues [43]. To determine where PLS3 function is required in *C. elegans* SMA models, we expressed PLS3 in either body wall muscle or neurons of *smn-1*(*cb131*) animals. Expression of human PLS3 in neurons (*rtEx852[unc-119p::PLS3]*), but not in muscles (*rtEx851[myo-3p::PLS3]*), restored normal recovery of locomotion rates after exhaustion in *smn-1*(*cb131*);*ChR2* animals (Fig. 2b, *p* = 0.05 and *p* = 0.237, versus *no PLS3*, respectively). This is consistent with studies of PLS3 and SMN in vertebrate models, where increasing PLS3 in neurons was also sufficient to ameliorate defects when SMN levels decreased [16].

Finally, we examined the impact of increased PLS3 activity in otherwise normal animals. A single-copy insertion of the ubiquitously expression *dpy-30p::PLS3* transgene had no impact on basal locomotion; activity in liquid was indistinguishable from control animals. However, further increase in PLS3 dosage did alter locomotion. A transgene generated at higher concentrations of *dpy-30p::PLS3*, *rtIs59*, increased basal locomotion rates by 38% (Fig. 2c, *p* = 0.003). However, it is unclear whether the increased locomotion of these high copy PLS3 overexpression animals is pertinent to the impact of PLS3 in SMA models. We cannot rule out that a high dosage of PLS3 is detrimental because it has been shown that high levels of the analogous actin-bundling protein Sac6 cause lethality in yeast [44]. Therefore, we avoided use of this high copy PLS3 transgene in the rest of this study, unless explicitly noted.

### Increasing plastin levels ameliorates NMJ and pharyngeal pumping defects caused by decreased *smn-1* function

*smn-1*(*cb131*) animals are resistant to paralysis by the acetylcholinesterase inhibitor, pyridostigmine bromide, suggesting reduced cholinergic NMJ function [35]. We found that diminished SMN-1 function also resulted in decreased sensitivity to aldicarb, another acetylcholinesterase inhibitor. Prolonged exposure to aldicarb gradually induces paralysis, due to acetylcholine accumulation in the synaptic cleft. *smn-1*(*cb131*) animals paralyze more slowly on aldicarb than control animals (Fig. 2d, log rank *p* = 0.009). Introduction of the human PLS3 multi-copy transgene into *smn-1*(*cb131*) animals restored normal aldicarb sensitivity (Fig. 2d, *p* = 0.009 versus transgenic control lacking PLS3). These results indicate that ubiquitous PLS3 expression improves cholinergic NMJ function when SMN-1 is impaired.

Various studies show that the effects of PLS3 may depend on the levels of SMN present. In models where SMN levels are substantially decreased, increased PLS3 insufficiently suppresses neuromuscular defects [15, 16]. However, to test if the beneficial impact of PLS3 is not limited to *smn-1*(*cb131*) animals, we also tested *smn-1*(*ok355*) severe loss of function animals, which contain even lower levels of SMN-1 activity compared to *smn-1*(*cb131*). *smn-1*(*ok355*) animals have decreased pharyngeal pumping rates, a behavior frequently used to assess neuromuscular function. *C. elegans* feed on bacteria using a dedicated set of muscles and neurons in the pharynx. Pharyngeal pumping rates average roughly 250 pumps per minute in the presence of food; late larval stage *smn-1*(*ok355*) animals have dramatically reduced pharyngeal pumping rates [31, 39]. In control animals with a wild-type *smn-1* gene, ubiquitous expression of human PLS3 in *C. elegans* slightly decreased pumping rates (single-copy transgene, *rtSi27*, − 11% versus wild type, Additional file 1: Fig. S1, *p* = 0.002). However, the introduction of the single-copy PLS3 transgene into *smn-1*(*ok355*) animals increased pharyngeal pumping rates by 50% (Additional file 1: Fig. S1, versus *ok355 p* = 0.011). Although pumping rates were not restored to wild-type levels, we conclude that in severe loss of function *smn-1* animals, increased PLS3 partially ameliorates neuromuscular defects. Using two different alleles and testing multiple defects, an overexpression of PLS3 proves beneficial when SMN levels are decreased in *C. elegans*. This concurs with the partial amelioration observed in particular type IIIb SMA patients that carry two *SMN2* copies and have elevated levels of PLS3 [12]. Taken together, our results demonstrate that plastin is a conserved genetic modifier of neuromuscular function.

### Identification of *sym-2* as a genetic modifier of both PLS3 and *smn-1*

**Fig. 3:**
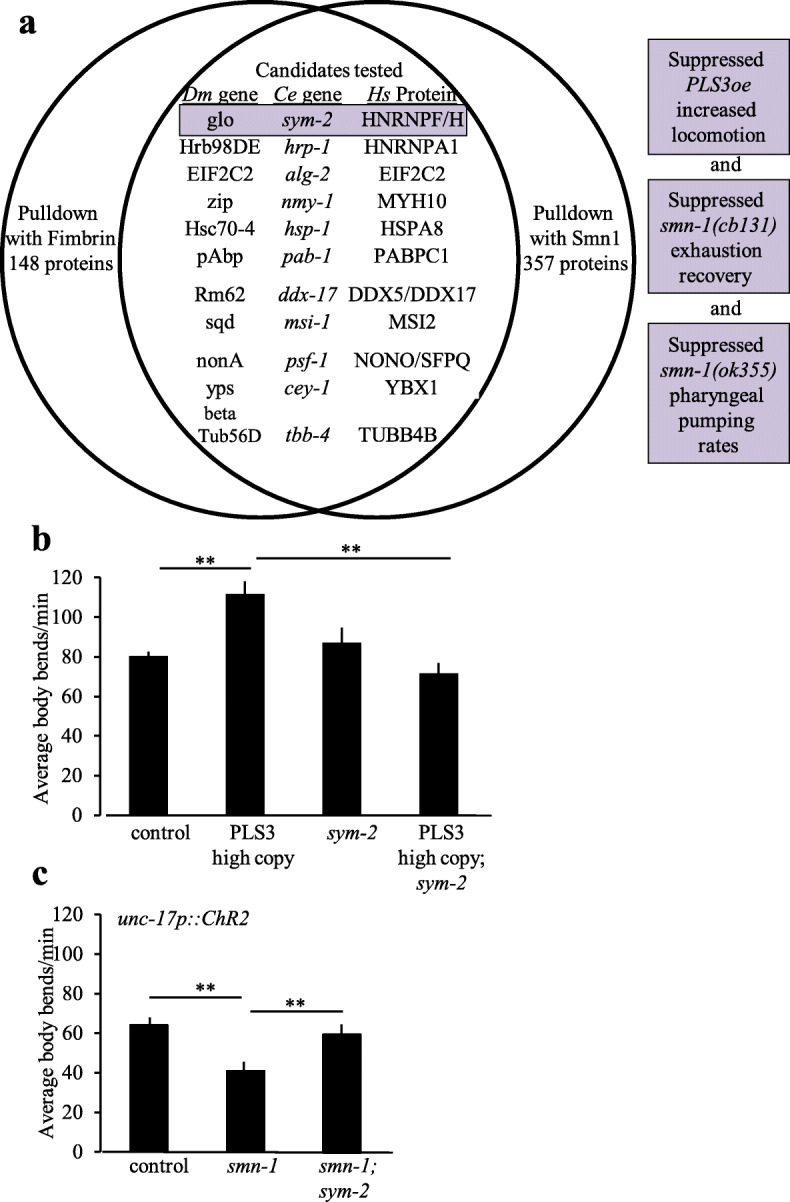
Identification of *sym-2* as a suppressor of *PLS3oe* and *smn-1* locomotion defects. **a** To find proteins in a putative SMN/PLS3 complex, we focused on candidates that independently pulled down in previous work [45] with both *Drosophila* Fimbrin and *Drosophila* Smn (left and right circles of Venn diagram, details in the “Methods” section). Candidates that had a corresponding *C. elegans* loss of function allele available in 2011 are listed and were examined. Only *sym-2* loss of function suppressed *PLS3oe* and *smn-1* defects in all three assays (right) used to screen these candidates. **b** Overexpression of human PLS3 increased basal locomotion rates compared to control animals. PLS3 was overexpressed from *rtIs59*, an integrated multi-copy transgene ubiquitously driving expression using the *dpy-30* promoter. Decreased function of the *C. elegans* hnRNP F/H ortholog, *sym-2*, did not change locomotion rates in a wild-type background, but *sym-2* perturbation suppressed increased locomotion in *PLS3oe* animals. ANOVA *F*(7962, 663) = 175.96, *p* < 0.05. **c** *sym-*2 perturbation suppressed locomotion defects in *smn-1*(*cb131*) animals immediately following ChR2 exhaustion. All animals in **c** carried *oxIs364[unc-17p::ChR2]*; ANOVA *F*(0.84, 0.55) = 17.51, *p* < 0.05. In all assays, *n* ≥ 30 animals per determination were used, combined from 3 independent trials. Scorers were blinded to the genotype of animals during the collection of data and data analysis. Student’s *t* test: **p < 0.01. SEM indicated

PLS3, SMN, and actin can be co-precipitated from HEK293T cells and murine spinal cord, but SMN does not directly interact with PLS3. The identity of other proteins in this complex is unknown [12]. Delineating other proteins in the PLS3/SMN complex would lead to a better understanding of SMA and the protective mechanism engaged by increased PLS3 levels. To identify these other proteins, we undertook a small, targeted screen starting with results from a previous large-scale proteomic study undertaken in *Drosophila* S2 cells [40]. From those results, we assembled a list of proteins that pulled down either with *Drosophila* SMN or with *Drosophila* Fimbrin, the PLS3 ortholog. We prioritized proteins found in both lists, based on frequency of pull-down; protein similarity between *D. melanogaster*, *H. sapiens*, and *C. elegans*; and the availability of *C. elegans* loss of function (*lf*) alleles. From this, eleven *C. elegans* candidate genes were targeted for functional analysis (Fig. 3a). We hypothesized that proteins in the same protein complex as SMN and PLS3 might show functional interactions, and we interrogated these putative interactions by testing if *C. elegans* candidate gene loss of function suppressed PLS3- and SMN-associated defects. High copy PLS3 expression results in aberrantly high locomotion levels in liquid (Figs. 2c and 3b). We determined if candidate gene loss of function alleles could suppress locomotion changes caused by increased PLS3.We also determined if candidate gene loss of function could suppress the defect in *smn-1*(*cb131*) recovery from ChR2-induced exhaustion. Only *sym-2* loss of function ameliorated defects in these assays (Fig. 3b, *p* = 0.007, and c, *p* = 0.006). RNAi knockdown of *sym-2* also suppressed *smn-1*(*cb131*) ChR2-induced exhaustion recovery defects (Additional file 1: Fig. S2, *p* = 0.02, *smn-1*(*cb131*) control versus knockdown of *sym-2*), but knockdown of genes encoding two related *C. elegans* proteins had no impact (*hrpf-1* and *hrpf-2*, Additional file 1: Fig. S2). Glorund was identified from the proteomic *Drosophila* screen, and BLAST results list the orthologs *sym-2*, *hrpf-1*, and *hrpf-2* in *C. elegans.* BLAST results also show the human orthologs to glorund as hNRNP F, H1, H2, GRSF1, and ESRP 1 and 2. Based on predicted functional domains and expression patterns, the human proteins most closely related to glorund and SYM-2 are hnRNP F, hnRNP H1, and hnRNP H2 [46]*.* Members of this heterogeneous nuclear ribonucleoprotein family have roles in pre-mRNA processing [42, 47, 48]. Given the results above, we chose to focus on *sym-2* for the remainder of this study.

*sym-2*(*mn617*) is a Y163N missense allele that likely decreases SYM-2 function [46]. As described above, introduction of *sym-2*(*mn617*) into the PLS3 overexpression background returned locomotion rates to normal (Fig. 3b, *p* = 0.007). And, introduction of *sym-2*(*mn617*) ameliorated *smn-1*(*cb131*) post-exhaustion locomotion defects in double mutant animals (Fig. 3c *smn-1*(*cb131*); *sym-2*(*mn617*) versus *smn-1*(*cb131*), *p* = 0.006). Taken together, these results demonstrate that decreased *sym-2* function suppresses defects caused by decreased *smn-1* activity or increased PLS3 function.

### PLS3 colocalizes with SMN and SYM-2 orthologs in vertebrate neurons

SMN-1, PLS3, and SYM-2 may act in a common functional pathway, but *C. elegans* genetic studies cannot determine if these proteins act in a conserved protein complex in mammalian neurons. Previously, SMN protein was found in RNP granules with various hnRNPs in neuronal processes, but not with hnRNP F/H orthologs [48–50]. While the majority of studies focus on the nuclear role in splicing of hnRNP F/H, one cannot exclude a possible role outside the nucleus [51, 52]. Therefore, a series of immunohistochemical assays were done to look for an association of hnRNP F/H with PLS3 and SMN that might be pertinent in cells and occur outside the nucleus.

**Fig. 4:**
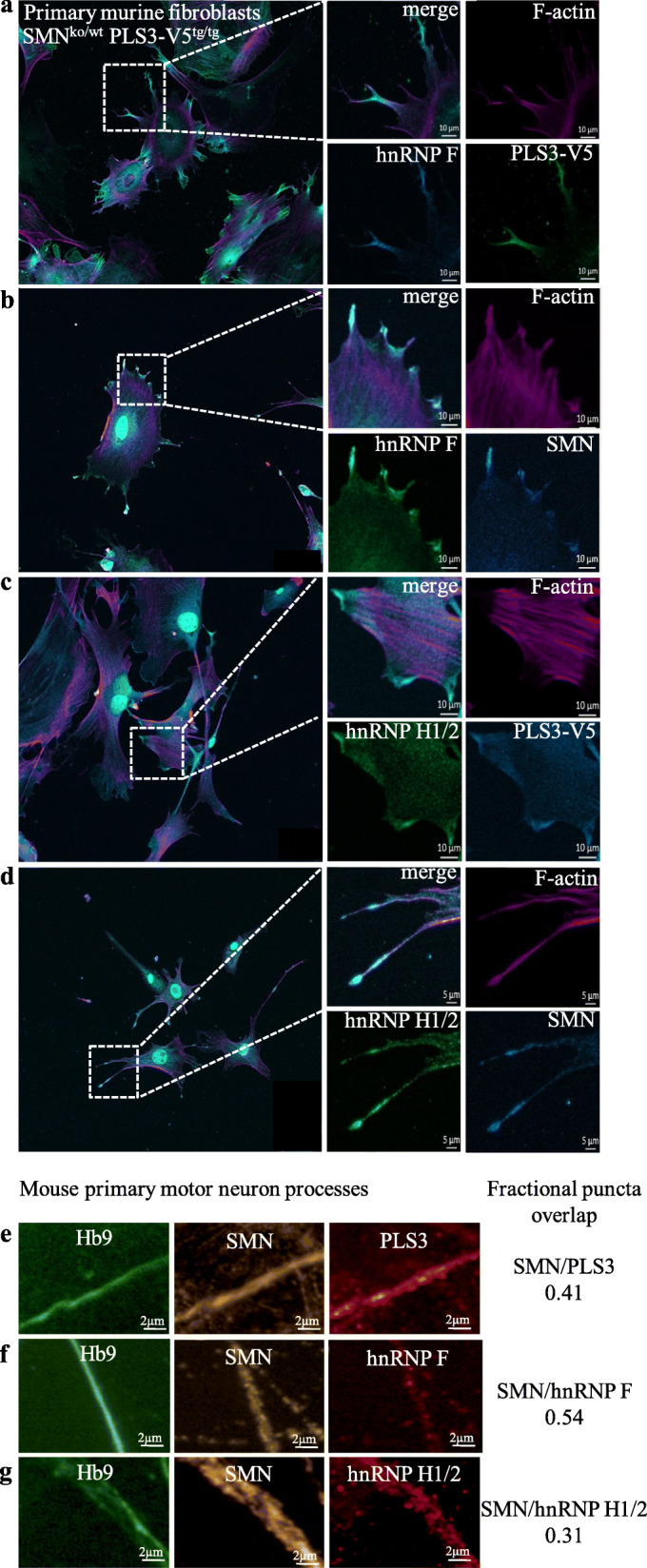
hnRNPF and hnRNPH1/2 localize with SMN and PLS3 in fibroblasts and motor neuron processes. Murine embryonic fibroblasts’ (MEFs) cell cultures were derived from *Smn* ^*+/−*^*; PLS3V5* ^*tg/tg*^ embryos and stained with V5 for PLS3 transgene; hnRNP-H1/2, hnRNP-F, and F-actin with phalloidin. **a** PLS3V5 localized to the same region as hnRNP F. **b** hnRNP F similarly localized with SMN. **c** hnRNP H1/2 localized to the same region with tagged PLS3. **d** hnRNP H1/2 localized to the same region with SMN. **e** SMN localized with PLS3 also in mouse spinal motor neurons processes in primary culture. Motor neurons were derived from GFP-Hb9-expressing embryos, which labels motor neurons. Endogenous protein was detected using immunohistochemistry. Quantitative analysis of cellular localization using confocal images of processes reports the percentage of localization for SMN fluorescence within processes. Protein fluorescence criterion was manually set using controls within the COLOMBUS software (Perkin Elmer), see the “Methods” section for details. **f** Localization of SMN with hnRNP F was seen using the same methods as described in **e**. **g** Localization of SMN with hnRNP H1/2 was seen using the same methods as described in **e**. Protein localization assays were done as three independent trials where each sample in each trial was a merge of images from multiple wells on a 96-well plate. Images were taken using an automated program, and then, slices were chosen incorporating planes with the nuclei

First, we examined localization of PLS3, SMN, hnRNP F, and hnRNP H1/2 in murine fibroblasts. Primary cultures were generated from mice overexpressing a V5-tagged PLS3 [16]. As expected, all four proteins were detected in fibroblast nuclei using immunohistochemistry. Interestingly, all four proteins are also present within cytoplasmic filopodia, suggesting they function in the same cellular compartment (Fig. 4a–d).

Next, we examined localization of hnRNP F, hnRNP H1/2, PLS3, and SMN in the processes of cultured mouse motor neurons. Embryonic stem cells were used to generate neuronal cultures from mice expressing motor neuron GFP (under the control of the Hb9 promoter). After 13 days of growth in culture, endogenous SMN, PLS3, hnRNP F, and hnRNP H1/2 proteins were detected within neuronal processes using immunohistochemistry. Each protein was distributed within motor neuron processes. To quantitatively assess protein localization within these structures, we used semi-automated image analysis software (Columbus, Perkin Elmer). By defining protein presence based on size, this analysis showed that SMN independently localized to the same region with all three proteins of interest in these structures (Fig. 4e–g), allowing us to conclude that hnRNP F and H1/2 are found in fibroblasts and in neuronal processes of motor neurons and that these proteins frequently localize to the same region with SMN. Collectively, these *cellular* data suggest that an association may happen between SMN, PLS3, hnRNP F, and H1/2 in vivo.

### SMN, PLS3, and SYM-2 orthologs co-exist in vertebrate protein complexes

**Fig. 5:**
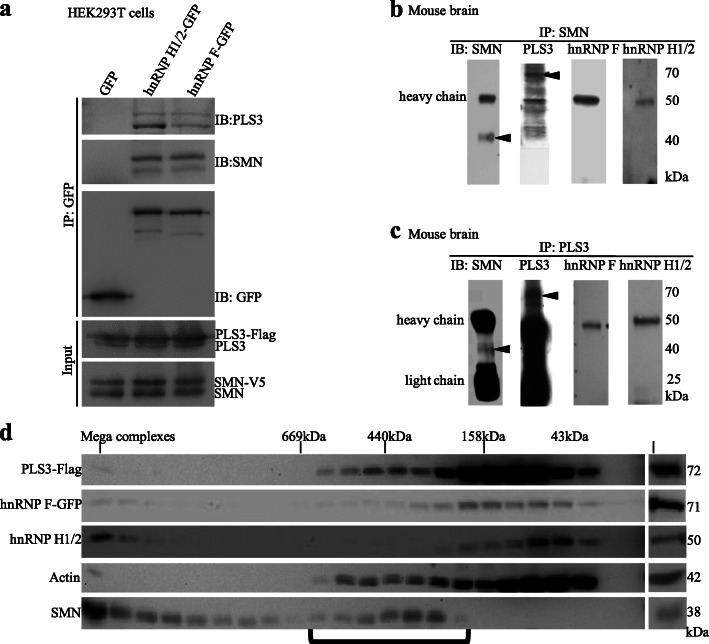
SMN, PLS3, hnRNP F, and hnRNP H1/2 associate in a large complex. **a** SMN and PLS3 independently associated with hnRNP H1/2 based on co-immunoprecipitation from HEK293T cells transfected with tagged proteins. **b** Endogenous PLS3, hnRNP F, and hnRNP H 1/2 associated with SMN in mouse brain extracts, based on immunoprecipitation with antisera against endogenous SMN protein. Arrowheads indicate the band of interest in blots with multiple bands. Heavy and light chain bands are labeled when present. **c** Endogenous SMN, hnRNP F, and hnRNP H1/2 associated with endogenous PLS3 using the same approach outlined in **b**. Arrowheads indicate the band of interest in blots with multiple bands. Heavy and light chain bands are labeled when present. IP on endogenous mouse brain extract was done from 3 independent brain extractions and preparations. **d** Size fractionation studies of whole cell lysates from HEK293T cells showed that actin, SMN, PLS3, and hnRNP F and/or H1/2 proteins co-fractionate in complexes of the indicated size (bracket), although the bulk of hnRNP F/H1/2 protein is in other smaller complexes. HEK293T cells were transfected with PLS3-Flag, and hnRNP F-GFP, endogenous hnRNP H1/2, actin, and SMN were detected using antisera

The localization analysis suggests that these proteins might be found in a similar cellular compartment and therefore could be in a complex together, a question we addressed using co-immunoprecipitation studies. HEK293T cells were transfected with tagged versions of all four proteins. Immunoprecipitation of GFP-tagged hnRNP F or hnRNP H1/2 revealed that these proteins are associated with both SMN and PLS3 (Fig. 5a). The interaction was not RNA-dependent, as associations were maintained after RNase treatment (Additional file 1: Fig. S3). These results are consistent with the localization analysis that show an interaction between the proteins.

Co-immunoprecipitation from mouse brain lysates confirmed an endogenous interaction between SMN and PLS3, hnRNP F, or hnRNP H1/2 (Fig. 5b bracket) in neuronal tissues. Reciprocally, endogenous PLS3 co-immunoprecipitated with SMN, hnRNP F, and hnRNP H1/2 (Fig. 5c). We conclude that SMN and PLS3 are in protein complexes that also contain hnRNP F and/or hnRNP H1/2 in the mouse brain. However, from this evidence alone, one cannot conclude that hnRNP F or hnRNP H1/2 are in a complex simultaneously containing both SMN and PLS3.

To test if these proteins are found in a complex together, we undertook size fractionation of whole cell lysates using FPLC columns and HEK293T cells expressing tagged versions of PLS3 and hnRNP F. In complexes of less than 669 kDa and greater than 158 kDa, four proteins—PLS3, SMN, actin, and hnRNP F—were found (Fig. 5d). And, in the smaller complexes within this range, hnRNP H1/2 also were found. Note that most of the PLS3, actin, and hnRNP F/H1/2 proteins are found in even smaller complexes, consistent with previously described interactions of these proteins. Taken together, size fractionation, co-immunoprecipitation, and localization results suggest that a rare previously unidentified complex may exist that contains actin, SMN, and PLS3 as well as hnRNP F and/or hnRNP H1/2.

### SYM-2 loss rescues *smn-1* endocytosis defects

**Fig. 6:**
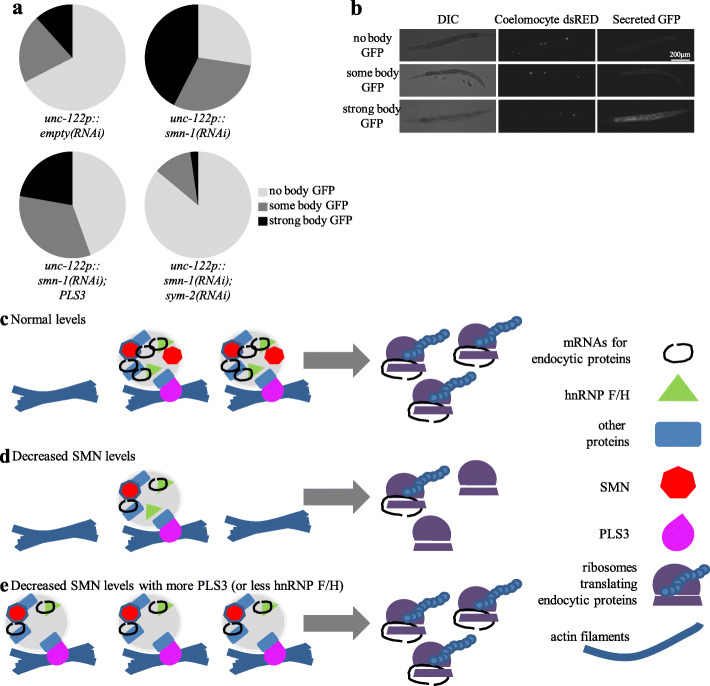
PLS3 overexpression and *sym-2*(*RNAi*) suppress endocytic defects caused by *smn-1*(*RNAi*) knockdown and summary. **a** Pie charts illustrating the percentage of animals with the indicated accumulation of GFP in the body cavity. Soluble GFP is secreted from the muscle cells into the body cavity. Coelomocyte-specific knockdown of *smn-1* leads to GFP accumulation in the body cavity due to endocytic pathway defects. Either coelomocyte-specific expression of PLS3 or coelomocyte-specific knockdown of *sym-2* suppressed GFP accumulation, consistent with cell-autonomous action of these proteins in coelomocytes. *n* > 40 animals pooled from multiple extrachromosomal array lines, combined from 3 independent trials. Scorers were blinded to the genotype of animals for each trial. Statistics calculated using chi-square. **b** Representative images for “no body GFP,” “some body GFP,” and “strong body GFP.” **c** Based on results presented here and previous work, a summary of functional genetic interactions is shown. **c** Normal levels of SMN protein facilitate appropriate transport and translation of “endocytic mRNAs,” which encode proteins involved in endocytosis. As an actin-bundling protein, PLS3 may contribute to transport or localization of RNP granules (gray circles) that contain “endocytic mRNAs,” as well as SMN, hnRNPF/H, and other proteins. **d** When SMN levels drop, the number, trafficking, or function of RNP granules containing “endocytic mRNAs” is perturbed, resulting in fewer endocytic proteins and endocytic defects. **e** The deleterious consequences of decreased SMN levels can be ameliorated by increasing PLS3 levels or by decreasing hnRNP F/H levels. The illustration of SMN and hnRNP F/H in an RNP tethered by PLS3 is speculative, but increasing PLS3 or decreasing hnRNPF/H ortholog activity ameliorates both functional defects and endocytic defects in the *C. elegans* model

Fluid-phase endocytosis is perturbed in *C. elegans* with decreased *smn-1* function [8]. For mechanistic insight in how *sym-2* loss suppresses *smn-1* defects, we used a classical *C. elegans* assay which assesses endocytosis in coelomocyte cells. Six coelomocyte cells are found in the body cavity (pseudocoelom) of adult *C. elegans*. These cells play a critical role in the active removal of proteins by endocytosis [53, 54]. When muscle cells in transgenic animals secrete soluble GFP into the pseudocoelom, GFP is taken up by coelomocytes through fluid-phase endocytosis and is sent for degradation. This assay has been used extensively to characterize endocytic pathways [55]. RNAi knockdown of *smn-1* in coelomocytes impairs GFP uptake and GFP clearance from the body cavity, consistent with endocytic pathway defects [8]. We found that either PLS3 overexpression or coelomocyte-specific RNAi knockdown of *sym-2* ameliorated endocytic defects in *smn-1*(*RNAi*) animals. Overexpression of PLS3 reduced the number of animals with GFP accumulation in the pseudocoelom (Fig. 6, *p* = 0.039), consistent with the previously demonstrated role of PLS3 in endocytic pathways [9]. Moreover, RNAi knockdown of *sym-2* dramatically improved GFP clearance from the pseudocoelom of *smn-1*(*RNAi*) mutant animals (Fig. 6, *p* = 0.1 × 10^−5^). These results demonstrate that the endocytic pathway defects observed when *smn-1* levels drop are counteracted by knockdown of *sym-2* in a cell autonomous manner. Combined with previous work, these results suggest that SMN, PLS3, and hnRNP F/H act in a protein complex, pertinent to SMA, whose function is required for normal function of endocytic pathways.

### PLS3 expression suppresses defects in a *C. elegans* ALS model

Increasing PLS3 levels accelerates neurite outgrowth in vertebrate motor neurons, regardless of SMN status, suggesting that increased PLS3 might impact general function and survival of motor neurons [12]. With the notion that common mechanisms may underlie neurodegenerative diseases, increased PLS3 expression may modify defects associated with other neurodegenerative diseases besides SMA [16, 20, 56]. Based on this hypothesis, we determined if increasing PLS3 levels would modify defects in an established *C. elegans* model of ALS [30].

In the previously characterized *C. elegans* ALS model, wild-type human SOD1 (SOD1-WT) or mutant human SOD1 carrying the patient amino acid change G85R (SOD1-G85R) are expressed at high levels using a pan-neuronal *C. elegans* promoter (from the *snb-1* gene). Mutant animals at young adult stage showed decreased locomotion in liquid, decreased pharyngeal pumping rates, and resistance to aldicarb, compared to their corresponding control animals (Additional file 1: Fig. S4, *p* = 6 × 10^−17^, *p* = 1.1 × 10^−8^, *p* = 6.6 × 10^−15^, *p* = 3.7 × 10^−10^, *p* = 4.7 × 10^–4^, *p* = 1.41 × 10^−8^) [30]. We introduced the single-copy PLS3 transgene *rtSi27* into both SOD1-WT and SOD1-G85R to determine if these defects would be suppressed.

Expression of PLS3 in *SOD1-G85R* animals proved to be beneficial. Locomotion rates increased 61% compared to *SOD1-G85R* animals lacking PLS3 (Additional file 1: Fig. S4a, *p* = 3 × 10^−6^). Pharyngeal pumping defects were similarly ameliorated; PLS3 expression increased pumping rates by 35% in *SOD1-G85R* animals, restoring pumping to normal levels (versus *SOD1-G85R* without PLS3, Additional file 1: Fig. S4b, *p* = 4.1 × 10^−8^). Sensitivity to paralysis by aldicarb was also restored in *SOD1-G85R* animals expressing additional PLS3, compared to *SOD1-G85R* animals without PLS3 (Additional file 1: Fig. S4c, *p* = 0.002). Overexpression of PLS3 does not indiscriminately increase locomotion or pumping, nor does it always change aldicarb sensitivity. *SOD1-WT* animals expressing PLS3 showed decreased locomotion rates, compared to *SOD1-WT* animals without PLS3 (Additional file 1: Fig. S4a, *p* = 1.7 × 10^−5^). PLS3 had no impact on pharyngeal pumping rates or aldicarb sensitivity in SOD1-WT animals (Additional file 1: Fig. S4b and 4c). Overexpression of SOD1-WT results in motor dysfunction, specifically in locomotion assays [57]. While we observed this defect as well, it is unclear why overexpression of PLS3 is detrimental to SOD1-WT animals when assessing locomotion but not for other behaviors. However, due to the consistent beneficial effects in mutant SOD1-G85R animals, we conclude that increased plastin levels can suppress defects in a *C. elegans* model of ALS.

To determine if PLS3 is beneficial outside of neurodegenerative disease models, we examined the impact of overexpression on animals with decreased locomotion due to synaptic defects. The *unc-25* gene encodes the *C. elegans* ortholog of mammalian GAD1 and is required for ɣ-aminobutyric acid (GABA) synthesis [58]. The behavioral defects of these animals include a 65% decrease in locomotion rates in liquid (Additional file 1: Fig. S5, *p* = 5 × 10^−25^ *unc-25*(*e156*) versus wild type). PLS3 expression did not increase locomotion rates (*p* = 0.576 versus *unc-25*). The *unc-13* and *unc-57* genes encode *C. elegans* orthologs of mammalian UNC13 proteins and Endophilin A protein, which are critical for presynaptic vesicular release and endocytosis, respectively. These mutant animals move in an uncoordinated manner compared to wild-type animals. PLS3 expression did not increase locomotion of animals lacking *unc-13* function, but did increase locomotion in animals lacking *unc-57* function (Additional file 1: Fig. S5, *p* = 0.02 *unc-57* versus *unc-57* with PLS3oe). Taken together, these results indicate that PLS3 overexpression is not generically beneficial and ameliorating endocytic pathways may improve function in *C. elegans* models of motor neuron disease.

### SYM-2 loss also improves locomotion in a *C. elegans* ALS model

Considering that PLS3 overexpression suppressed defects in a *C. elegans* disease model of ALS and the relationship between PLS3 and SYM-2 described above, it seemed plausible that *sym-2* might also be a modifier in an ALS model. We assessed the impact of *sym-2* on locomotion in the *C. elegans* SOD1 overexpression model. Using RNAi to knockdown *sym-2* levels, *SOD1-G85R* animals had increased locomotion rates with *sym-2*(*RNAi*) compared to empty vector RNAi feeding (Additional file 1: Fig. S4d, versus *control* (*RNAi*), *p* = 3.7 × 10^−8^). These results suggest that, reminiscent of PLS3, the beneficial impact of hnRNP F/H loss and endocytic pathway changes may extend to other motor neuron diseases.

## Discussion

Elucidating how the pathology and symptoms of motor neuron diseases can be suppressed should yield important insights into neurodegenerative mechanisms. Here, we focused on understanding functional interactions between PLS3 and SMN, and on how their interaction provides insight into pathways pertinent to disease. We confirm that increasing PLS3 suppresses locomotion defects and endocytic pathway defects that are caused by decreased SMN levels. In addition, we identify hnRNP F/H as a component of protein complexes containing SMN and PLS3. We demonstrate that decreasing levels of the *C. elegans* hnRNP F/H ortholog SYM-2 also suppresses both locomotion and endocytic pathway defects caused by diminished *smn-1* function. Additionally, we find that the beneficial impact of increased PLS3 or decreased SYM-2 expression is not limited to SMA; defects in a *C. elegans* model of ALS are also suppressed. Finally, we show that interactions identified in *C. elegans* are likely conserved, as PLS3, SMN, hnRNP F, and/or hnRNP H1/2 are found together in mammalian neuronal tissues and motor neuron processes. Combined, these results suggest that SMN and PLS3 act to ameliorate SMA defects via protein complexes that contain hnRNP F and/or H1/2 that is pertinent to endocytic pathways.

Using mouse fibroblast cells and primary motor neurons, we demonstrated that PLS3, SMN, hnRNP F, and hnRNP H1/2 have an association and localize to the same regions in vitro. Biochemical analysis confirmed these findings in HEK293T cells and mouse brain extracts. Additionally, fractionation studies from mouse neuronal tissues and/or HEK293T cells suggested that PLS3, SMN, and hnRNP F and/or H1/2 proteins can be found in protein complexes. Given the biochemical interactions identified in this study, the genetic interactions observed between SMN-1, PLS3, and SYM-2 likely also exist between the vertebrate orthologs SMN, PLS3, and hnRNP F/H1/H2. Discovering how hnRNP F/H family proteins interact with SMN and PLS3 will yield insights into aspects of their function critical in disease. hnRNPs are a diverse group of RNA binding proteins originally named for their ability to bind hnRNAs (heterogeneous nuclear ribonucleic acids) [47]. hnRNP proteins have diverse roles and are most commonly involved in pre-mRNA processing, including mRNA export, localization, translation, and stability [42]. While studies have identified that altering hnRNP F/H expression affects proliferation of cancer cells, virtually nothing is known about their role or what altering hnRNP F/H expression levels would do in a neurological context. Here, we find that loss of SYM-2 suppresses endocytic pathway defects in animals with decreased SMN-1 activity, identifying a new role for this specific hnRNP sub-family. Understanding the function of hnRNPs beyond their canonical role and identifying how they impact endocytic pathways may lead to a better understanding of endocytosis and SMA.

In *C. elegans*, mouse, and zebrafish models of SMA, endocytic defects are seen at the NMJ [8–10]. In Huntington’s and ALS models, clathrin-mediated endocytosis is perturbed, potentially by protein aggregates that compete with clathrin for chaperone proteins [59]. Furthermore, mutations in CHMP2B, part of a complex involved in endosome-lysosome fusion, cause frontotemporal dementia [60]. Given these commonalities, we speculate that perturbation of endocytic pathways may be a critical step in neurodegenerative disease pathology. Using genetic and functional studies, we showed increased PLS3 expression or decreased SYM-2 expression ameliorated endocytic pathway defects in a model of SMA. This data reinforces that endocytic pathway defects are critical in motor neuron disease and further highlights the importance of understanding why these hnRNPs are important in endocytosis.

Why does increasing PLS3 expression or decreasing SYM-2 expression ameliorate behavioral defects in models of SMA or ALS? Our results demonstrate that these proteins colocalize with SMN, in motor neuron processes. But how does this connect motor neuron disease to endocytic pathways? Previous work has shown that increased PLS3 does not increase overall SMN protein levels [12, 15]. However, increased PLS3 may lead to stabilization or transport of specific RNP complexes that are disrupted when SMN levels decrease. Previous work also demonstrated that a minimal amount of SMN is required for PLS3 to ameliorate symptoms, consistent with the more dramatic impact of PLS3 overexpression on *smn-1*(*cb131*) partial loss of function versus *smn-1*(*ok355*) complete loss of function animals. Previous studies have also analyzed animals with decreased plastin levels. In *smn* mutant zebrafish, PLS3 protein levels were found to correspondingly decrease when SMN levels decreased. However, this correlation was not repeated when profilin II levels were looked at, thus suggesting the interaction with PLS3 is unique [38].

Using the results presented herein along with previously published work, we suggest a model in which the balanced and coordinated action of SMN, PLS3, and hnRNPF/H proteins promotes the optimal transport and/or function of mRNAs and their encoded proteins, and further that these mRNAs and proteins are essential for normal endocytic function (Fig. 6). We suggest that decreased levels of SMN negatively impact mRNAs required for endocytosis and that appropriate increased expression of PLS3 or decreased expression of hnRNP F/H levels can counteract these negative impacts. As summarized in Fig. 6, decreased SMN protein levels may lead to decreased function or transport in axons of RNP granules containing hnRNP F/H. However, increasing PLS3 leads to improved RNP granule transport or function, despite diminished SMN levels. Decreasing hnRNP F/H may improve the translation and/or function of endocytic proteins encoded by mRNAs within these granules. In either case, increasing PLS3 or decreasing hnRNP F/H improves endocytic function, which partially ameliorates the deleterious consequences of diminished SMN function.

## Conclusions

Genetic studies are important in understanding disease pathology and can yield novel and unexpected insights into disease mechanisms. This is exemplified by the identification of PLS3 and NCALD as modifiers of SMA in patient families and the identification of hnRNP F/H as a genetic modifier herein. Based on the level of conservation observed, it seems likely that decreasing hnRNP F/H protein function would also be beneficial in vertebrate SMA models, and potentially in ALS models as well. The identification and functional interaction identified between SMN, PLS3, and hnRNP F/H provide insight into the mechanisms that lead to perturbations in motor neuron disease.

## Methods

*C. elegans* strains were maintained at 20 °C under standard conditions (Additional file 1: Table 1) [61]. All animals in experiments with *smn-1*(*ok355*) were first generation progeny of heterozygous for the *hT2* balancer to preserve a uniform genetic background. RNAi studies were undertaken in a sensitized background containing the transgene, *uIs72*, that expresses the SID-1 double-stranded RNA channel in neurons [62]. RNAi clones were verified by sequencing and aligned for specificity to the gene of interest. Only one RNAi clone was tested for each gene of interest; however, in the case of *smn-1* and *sym-2*, loss of function alleles showed the same phenotype in behavioral testing. For behavioral assays with transgenic animals, at least 3 integration lines were tested to confirm results and rule out behavioral phenotypes due to the integration site. All integrated transgenes were backcrossed to wild type at least three times before final strain construction. The transgenic strains *rtSi27* and *rtSi28* were generated by Mos-mediated single-copy insertion [63]. The *dpy-30* promoter was cloned into pPD49.26 from the *C. elegans* expression vector pS235, containing the *dpy-30* promoter via SphI and NheI sites. Human PLS3 CDS (ATG to TAA) was amplified from pcDNA3.1_PLS3.V5-His6 TOPO vector [9] using primers 5′-GAACGCTAGCATGGATGAGATGGCTACCAC-3′ and 5′-CAGGGGAATGAAGAGAGTGTAACCCGGGGTTC-3′ and cloned behind the *dpy-30* promoter creating *dpy-30p::PLS3*. Either the promoter alone or the promoter with the PLS3 coding sequence was cloned into a modified pCFJ66 via SphI and NheI or SphI and XmaI sites, respectively. The final constructs pHA#606 *dpy-30p::unc-54UTR*, *unc-119*(+) and pHA#607 *dpy-30p::PLS3::unc-54UTR*, *unc-119*(+) were injected at 50 ng/μl with the standard MosSCI cocktail and injected into EG4322 (*unc-119(ed3)III*;*cxTi10816 IV*) animals. Single-copy insertion events were identified based on rescue of *unc-119* in non-fluorescent animals and confirmed by PCR genotyping. Transgenic strains for neuronal and muscle-specific rescue were generated by cloning the *unc-119* or *myo-3* promoters using SphI and NheI sites into pHA#606 and pHA#607, in place of the *dpy-30* promoter. Both single-copy and multi-copy transgenes were generated by injection of 50 ng/μl of targeting plasmid into EG4322 (*unc-119(ed3)III*;*cxTi10816 IV*) animals. Multi-copy insertion events were identified based on the rescue of *unc-119* in animals expressing fluorescent markers. *Ceplst-1* overexpression animals were created by amplifying *plst-1* from the yk1465f08 clone using primers 5′-GGTACCGGTTTAATTACCCAAGTTTG-3′ and 5′-GAGCTCCAGAAAAACCGAAAAAATCC-3′. The cDNA was placed behind the *dpy-30p* in pPD49.26 using KpnI and SacI restriction sites. Either promoter alone or promoter with *plst-1* coding sequence was injected in *smn-1*(*cb131*)*;*ChR2 animals at 30 ng/μl along with the co-injection marker pJM#67 *elt-2p::GFP* [64].

Coelomocyte plasmids were created by cloning either PLS3 cDNA or *sym-2* RNAi into the *unc122p::smn-1*(*RNAi*) plasmid using AgeI and NotI. Primers for amplification of *sym-2* RNAi were as follows: 5′-gaacGAATTCTGAGAGGATTGCCTTATGATTGT-3′ and 5′-gaacACCGGTctcgatCTATTCCATCAAAATCG-3′. Plasmids were injected into GS1912 at 40 ng/μl alongside *coel::RFP* (Addgene #8938) also at 40 ng/μl. Transgenic lines tested are listed in Additional file 1: Table 1.

### Selection of genes for analysis

To find proteins in a putative SMN/PLS3 complex, we drew on published pull-down studies in *Drosophila* S2 cells [45]. We identified proteins that (i) independently pulled down with Fimbrin or Smn with an HG score of more than 61.5 (< 0.05 false discovery rate) consistent with “strongly predicted interaction,” (ii) were not in the top 10% of proteins pulled down in the entire database (to avoid non-specific interactions), and (iii) had a *C. elegans* loss of function allele available in 2011.

### *C. elegans* assays

Retinal feeding was undertaken as previously described [37] with modifications noted below. Transgenic animals were cultivated in the dark at 20 °C on NGM plates with OP50 bacteria with or without all-trans retinal (Sigma), for approximately 18 h before experiments. Plates containing all-trans retinal were prepared by spreading 100 μl of OP50 culture mixed with 1.6 μl of 100 mM all-trans retinal stock (in ethanol) onto 2.5-cm plates containing 5 ml of NGM. After the animals were picked to individual wells containing PBS and given 5 min to acclimate to the environment, pre-blue light exposure videos were taken using an AxioCam R3.0 camera at 12 frames/second, on a Zeiss SteREO Discovery V20 microscope at × 137.5 magnification. Immediately following the initial video, animals were illuminated with a mercury bulb and a Zeiss bandpass filter set to 470 nm to excite channelrhodopsin for 20 s. In order to maintain consistent blue light dosage, light intensity was measured weekly using a spectrometer. To ensure all animals were equally exposed to blue light, a magnification was chosen where the blue light field illuminated well beyond the animals being studied. Post-exhaustion videos were then taken immediately following blue light exposure.

Locomotion analysis was done with young adult animals that were individually picked into a single well of a PDMS chip. Wells within the chip have a diameter of 1.6 mm and depth of 0.15 mm. Each well was preloaded with 2 μl of PBS, and assays were performed at 22 °C. After animals were placed in the wells, they were left undisturbed for 5 min to allow for acclimation to the environment. Locomotion rate was then assessed by taking 10-s videos at relevant time points with an AxioCam R3.0 camera at 12 frames/second, on a Zeiss SteREO Discovery V20 microscope at × 137.5 magnification. Movies were analyzed using the NABAS software, modified for image formats, and then translated to body bends per minute [65]. A body bend is defined as a muscle contraction resulting in a dorsal to ventral movement across the midline of the animal. Average body bends per minute (± SEM) were combined from at least three independent trials (*n* ≥ 30 animals in total).

Pharyngeal pumping analysis was performed in the last larval stage or in young adults as previously described [39]. Pharyngeal grinder movement in any axis was scored as a pumping event. Average pumping rates (± SEM) were combined from at least three independent trials (*n* ≥ 30 animals in total).

For locomotion studies, RNAi by feeding was done on second generation (F2) animals reared on either control (p*L4440*) or experimental, *sym-2* [46], *hrpf-1*, or *hrpf-2* dsRNA feeding vectors in HT115 bacteria and assayed. The *sym-2* RNAi clone contains genomic DNA inserted into the pL4440 vector, corresponding to the last 3 exons and the 3′UTR of the gene, amplified by primers 5′-CTCGATCTATTCCATCAAAATCG and 5′-TGAGAGGATTGCCTTATGATTGT [66]. *hrpf-1* and *hrpf-2* RNAi clones were made by amplifying from genomic DNA using the following primers, respectively, and inserted into the pL4440 vector (5′-ATGGGAGGCCACTGAACAGG and 5′-GTGATCCATACGCCGCAGAAT; 5′-CGCCCACTGATGTCCGTGG and 5′-GCTGGAGATCATCGAAGTGG). RNAi clones were verified by sequencing and aligned for specificity to the gene of interest.

Aldicarb assays were conducted as described [67] with noted modifications. Aldicarb (Sigma-Aldrich) was added to NGM at a final concentration of 1 mM. Thirty microliters of OP50 bacteria was seeded in the center of the plates on the day of the assay. The plates were allowed to dry without lids for at least 20 min. Approximately 15 young adult animals were transferred with a platinum wire onto an aldicarb assay plate. The animals were examined every hour and scored as paralyzed if they did not pump or move after being prodded with a platinum wire. The experimenter was blinded to the genotypes/treatment. Average fraction of paralyzed animals (± SEM) was obtained by combining at least 3 independent trials (*n* ≥ 30 animals in total).

Coelomocyte endocytic assays were undertaken as described previously [8]. Young adult animals were scored as strong-intense GFP (body cavity filled), some-weak GFP (little GFP in body cavity), or none-no GFP (no GFP visible in the body cavity). Only animals expressing the array in all 6 coelomocytes were scored (determined by the presence of the coelomocyte specific RFP co-injection marker) by an observer blinded to RNAi treatment. Averages were determined over 3 independent trials with an *n* > 40.

For the dispersal assay, 60 × 15 mm NGM plates were seeded with 200 μl of OP50 *E. coli* and evenly spread over the entire surface. Three rings were drawn on the bottom of the plate, starting 5 mm from center and every 5 mm out towards the edge. Strains were maintained at 20 °C for the duration of the experiment. Approximately 24–32 young adult worms were manually placed in the center of the plate and moved back to 20 °C. Every 60 min for 180 min, the strains were counted and categorized based on which ring they were in. Animals that moved outside the 3rd ring were counted for total worm count. Data is represented as fractional percent of animals located in each ring.

### Preparation of primary murine embryonic fibroblasts (MEFs), cell culture, and transfection

Primary murine fibroblasts were prepared from E13-14 animals as previously described [16]. Fibroblasts were isolated from *Smn*^*ko/wt*^*;PLS3V5*^*tg/tg*^ embryos [9].

HEK293T cells and MEFs were grown in culture medium (Dulbecco’s modified Eagle’s medium (DMEM) supplemented with 10% fetal calf serum (FCS), 1% penicillin/streptomycin, and 0.1% amphotericin) at 37 °C and 5% CO_2_. Eighty percent confluent HEK293T cells were transiently co-transfected with the respective vectors using Lipofectamine 2000 (Invitrogen) and cultured in OptiMEM. Fresh culture medium was added 6–12 h post-transfection to the transfected cells. Cells were harvested 48 h post-transfection in PBS, and cell pellets were stored at − 80 °C.

### Immunofluorescence, immunoblotting, and antibodies for MEFs and HEK293T cells

For immunofluorescent staining, MEFs were fixed with 4% paraformaldehyde in PBS for 15–20 min. Fibroblasts were permeabilized in 0.2% Triton X-100 in PBS for 5 min; blocked for 45 min in 5% FCS, 5% bovine serum albumin (BSA) in PBST; incubated overnight at 4 °C with the primary antibodies (1:50) in blocking solution; washed with PBS; and incubated with the secondary antibodies (1:200) and phalloidin (1/40) for 3–4 h. For immunofluorescent staining, the following secondary antibodies were used: AlexaFluor 488 (Do anti-Ms 1:300, or Do anti-Rb, 1:300), AlexaFluor 350 (Do anti-Ms 1:300, or Do anti-Rb, 1:300), and phalloidin AlexaFluor 568 (Invitrogen, A12380). Cells were imaged using a fluorescence microscope (equipped with an ApoTome (AxioImager M2, Zeiss)). For immunoblotting, the following primary antibodies with the listed concentrations were used: mouse anti-V5 (Invitrogen, R960-25, 1:1000), rabbit anti-hnRNP H2 (Novus Biologicals, NBP1-89816, 1:500), rabbit anti-hnRNP F (Novus Biologicals, NBP1-57273, 1:500), rabbit anti-PLS3 ([12] 1:1000), anti-SMN (BD Transduction Laboratories, 610647, 1:3000), and anti-GFP (produced in hybridoma cell lines, 1:250). For signal detection, the HRP-conjugated secondary antibodies goat anti-mouse (Sigma, A4416, 1:5000) and goat anti-rabbit (Cell Signalling, 7074, 1:5000) lgG were used along with the SuperSignal West Pico Chemiluminescent Substrate (Thermo Scientific). For reprobing, the membranes were stripped for 10 min with the Restore Western Blot Stripping Buffer (Thermo Scientific).

### Plasmids

Human *hnRNP F* (NM 001098208.1) and *hnRNP H2* (NM 019597.4) cDNA was cloned into pcDNA3.1/CT-GFP vector and human *SMN1* (NM 000344.3) was cloned into pcDNA3.1/V5-His vector via TOPO TA cloning as described in the manufacturer’s protocol (Invitrogen). Human *PLS3* (NM 005032.6) cDNA was cloned into pcDNA-Flag via MluI and NotI sites. The pcDNA3.1/CT-GFP-TOPO vector (Invitrogen) was used as a negative control for the co-immunoprecipitations.

### Co-immunoprecipitation in HEK293T cells

For GFP-immunoprecipitation (IP), GFP MicroBeads, μ-columns, and μMACS Separator (Miltenyi Biotec) were used. μ-columns were permeabilized with 10% Triton X-100 and washed twice with IP lysis buffer (100 mM NaCl, 50 mM Tris, 5 mM EGTA, 1% CHAPS, pH 7.5, containing protease inhibitor). Transfected HEK293T cells were lysed for 20 min on ice in IP lysis buffer, sonicated for 10 min, and centrifuged for 20 min at 13,000 rpm at 4 °C. Next, 30 μl of GFP MicroBeads was added to 500 μl cell lysate and incubated for 2 h at 4 °C. After adding the cell lysates to the μ-columns, the μ-columns were washed 5 times with IP lysis buffer, and proteins were eluted from the μ-column using hot (95 °C) SDS sample buffer. For RNase treatment, 100 μg/ml RNase A (Invitrogen) was added to the IP lysis buffer. Eluates were analyzed by SDS-PAGE and subsequent Western blot.

### Mouse motor neuron differentiation

Mouse wild-type hb9::GFP ES colonies were differentiated into motor neurons during 6 days and dissociated at day 7, using an embryoid body method as described previously [68]. Briefly, after 2 days, EBs were treated with retinoic acid (RAc; 100 nM, Sigma-Aldrich) and a Hedgehog agonist (500 nM, Curis Inc.). On day 3, EBs were treated with RAc (100 nM) and the Hedgehog agonist (1 μM) and incubated for 96 h. On day 7, papain (Worthington Biochemical) and DNase I were used to dissociate EBs. Motor neurons were then dissociated, plated into poly-ornithine coated greiner 96-well plates (cat #655090) at a density of 20,000 cells per well, and cultured in DMEM-F12 medium (Invitrogen) containing 2% FBS (Invitrogen), B-27 supplement (Invitrogen), 20 ng/ml GDNF and BDNF, CNTF (R&D Systems), insulin, progesterone, BSA, selenite, and apotransferrin (Sigma-Aldrich).

### Immunocytochemistry of primary motor neurons

Day 13 motor neuron cultures were fixed using 8% PFA for 15 min, followed by permeabilization and blocking using 0.25% Triton X-100 and 10% normal goat serum in PBS for 30 min. Detection of endogenous proteins was performed using the following antibodies: mouse anti-SMN antibody (BD Transduction Laboratories, diluted 1:200), rabbit anti-SMN antibody (Santa Cruz, diluted 1:200), mouse anti-PLS3 antibody (Santa Cruz, diluted 1:500), rabbit anti-hnRNP H antibody (One World Lab, diluted 1:1000), and rabbit anti-hnRNP F antibody (Abcam, diluted 1:500). Secondary antibodies for detection included AlexaFluor 546 (Gt anti-Rb, 1:300, or Gt anti-Ms, 1:300) and AlexaFluor 647 (Gt anti-Rb, 1:300).

### Localization analysis

Images were taken on an Opera system (Perkin Elmer) using a 40x water immersion lens with laser excitation at 405, 488, 561 and 635 nM and used for analysis. Confocal stacks were taken using a Nipkow spinning disk at 1 μm slice intervals. Images were stacked together and analyzed for localization of antibody staining using Perkin Elmer’s Columbus program (http://www.perkinelmer.com/pages/020/cellularimaging/products/columbus.xhtml).

### Co-immunoprecipitation (Co-IP) of mouse brain lysate

Whole adult mouse brains of Gad2-EGFP mice were prepared by homogenization, using a Polytron homogenizer in ~ 20 ml of cold 1× RIPA buffer and then incubated on ice for 30 min. Supernatant was collected after a 20-min centrifuge step at 13,500 rpm in 4 °C and stored at − 80 °C. Protein concentrations were determined using the BSA Protein Assay kit (Bradford).

Seven hundred fifty micrograms of lysate was incubated with 2 μg of antibody for 1 h on a rotator at 4 °C. During incubation, protein G beads were washed 5 times in PBS and final bead suspension was added to lysate mixture and incubated for 3 h on a rotator at 4 °C. The bead mixture was centrifuged down and washed three times, removing all supernatant. The beads were then boiled in 2× SDS loading dye at 95 °C for 5 min to elute the proteins. Eluted proteins were run on an SDS-polyacrylamide gel for Western analysis.

### Western analysis

Equal amounts of protein preparations were run and separated on a 10% SDS-polyacrylamide gel electrophoresis and transferred to a PVDF membrane. Membranes were blocked with 5% milk in Tris-buffered saline containing 0.1% Tween 20 (TBST) overnight at 4 °C. They were probed with the antibody for 1 h at RT at the following dilutions: mouse anti-SMN antibody (BD Transduction Laboratories, diluted 1:2500), mouse anti-PLS3 antibody (Santa Cruz, diluted 1:1000), rabbit anti-hnRNP-H antibody (One World Lab, diluted 1:10,000), and rabbit anti-hnRNP F antibody (Abcam, diluted 1:2500). After washing with TBST, membranes were incubated with HRP-conjugated secondary antibodies (anti-mouse or anti-rabbit immunoglobulin-horseradish peroxidase, both at 1:2500 dilution) at RT for 30 min, and the blots were developed using ECL and visualized on autoradiography films. The presence of protein was determined based on the size of the band compared to a ladder.

### Size exclusion chromatography (SEC)

HEK293T cells were transfected with hnRNP F-GFP and Flag/His-PLS3 vectors. The size exclusion chromatography was performed on a Superose 6, 10/300 GL column (GE Healthcare) connected to the FPLC system. The SEC column was calibrated with gel filtration standard proteins including thyroglobulin (669 kDa), ferritin (440 kDa), aldolase (158 kDa), ovalbumin (43 kDa), carbonic anhydrase (29 kDa), and ribonuclease A (13.7 kDa). To solubilize proteins including membrane fraction, cells were lysed in the lysis buffer (1% NP40, 50 mM Tris-HCl (pH 7.4), 150 mM NaCl, and 2 mM MgCl2, containing protease inhibitor cocktail). Lysis buffer without protease inhibitor was degassed and used for column equilibration. The cell lysate was sonicated and centrifuged at 13,000 rpm for 15 min. Two hundred fifty microliters of cell lysate was injected into the column. Fractions of 0.5 ml were collected and frozen at − 80 °C. Twenty-five microliters of each fraction was analyzed by Western blotting.

## Supplementary information

**Additional file 1: Figure S1.** PLS3 overexpression suppressed pharyngeal pumping defects in *smn-1*(*ok355*) animals. Homozygous null *smn-1*(*ok355*) animals lacking *smn-1* survive through early larval stages due to maternal loading of SMN-1 proteins and mRNA by heterozygous *smn-1*(*ok355*)*/hT2* mothers. *hT2* carries a functional copy of endogenous *smn-1*, overexpression of human PLS3 slightly lowered pumping rates in control *smn-1*(+) animals, but increased pumping rates in homozygous *smn-1*(*ok355*) animals (compared to control *smn-1*(*ok355*))*.* To control for genetic background all animals were derived from mothers heterozygous for *hT2*. To control for transgene insertion position, control and *smn-1*(*ok355*) animals carried *rtSi28 [dpy-30p::empty].* PLS3 is overexpressed from *rtSi27 [dpy-30p::PLS3]*, a single copy insertion on chromosome II. *n*≥30 animals per determination, combined from 3 independent trials that the scorer was blinded to the genotype of animals. ANOVA *F*(9.9,13.1) = 14.23, *p<0.001*; post-hoc Mann-Whitney U-test *p<0.05, S.E. indicated. **Figure S2.** *sym-2* knockdown using RNAi suppressed the *smn-1* locomotion defect. Exhaustion of cholinergic motor neurons using *ChR2* slowed locomotion; *smn-1*(*cb131*) animals with decreased SMN-1 function had aberrantly low locomotion rates post-exhaustion. RNAi knockdown of hnRNP F/H ortholog *sym-2* ameliorated this defect. *empty* (*RNAi*) used as a control as the bacterial strain used for RNAi can alter locomotion rates. n≥30 animals per determination, combined from 3 independent trials. Student’s *t*-test *p<0.05 S.E.M. indicated. **Figure S3.** Association of SMN and PLS3 with hnRNPF or hnRNPH1/2, is not dependent on RNA. Pretreatment with RNase had no impact on co-immunoprecipitation of PLS3 or SMN from HEK293T cells using GFP tagged hnRNP F or hnRNP H1/2. SMN was tagged with V5; PLS3 was tagged with Flag. Conditions and procedures as described in Fig. 4a. **Figure S4.**PLS3 and *sym-2* suppress behavioral defects in specific *C. elegans* models of neurodegenerative disease. (a) Neuronal overexpression of wild type human SOD1 (SOD1-WT) had little impact on *C. elegans* locomotion, but overexpression of SOD1 carrying the familial ALS patient allele G85R (SOD1-G85R) dramatically decreased locomotion in young adult animals. Introduction of ubiquitously expressed human PLS3 decreased locomotion rates in *SOD1-WT* animals, but ameliorated defects in *SOD1-G85R* animals. ANOVA *F*(320.2,967.5) = 6.78, *p<0.05* (b) *SOD1-G85R* animals had decreased pharyngeal pumping rates in young adult animals. PLS3 expression restored normal pumping rates in *SOD1-G85R* animals. ANOVA *F*(1305379,327194) = 235.38, *p<0.001* (c) *SOD1-G85R* mutant animals were resistant to the cholinesterase inhibitor aldicarb, based on slower time to paralysis. Introduction of ubiquitously expressed human PLS3 restored sensitivity to normal levels. (d) RNAi knockdown of *sym-2* had no impact on *SOD1-WT* animals, but increased locomotion rates in *SOD1-G85R* animals. Empty RNAi was used as a control for *sym-2*(*RNAi*) as bacterial strain can alter locomotion and other behaviors. *sym-2*(*RNAi*) had no impact on *SOD1-G85R* pumping rates (not shown). In this figure PLS3 was ubiquitously expressed from *rtSi27 [dpy-30p::PLS3]*, a single copy insertion on chromosome II. In each assay an *n*≥30 animals per determination, combined from 3 independent trials. In all trials scorers were blinded to the genotype of the animals when collecting and scoring the data. Mann-Whitney U-test (panels a,b,d,e and f) or Log Rank test (panels c and g) **p<0.01 S.E.M. indicated. **Figure S5.** PLS3 does not generically suppress locomotion. (a) *C. elegans* lacking glutamic acid decarboxylase encoded by *unc-25* had decreased locomotion in liquid due to defective GABA synthesis (Jin et al., 1999). The ubiquitous expression of PLS3 did not suppress locomotion defects in *unc-25;PLS3oe* double mutant animals. PLS3 was overexpressed from *rtSi27 [dpy-30p::PLS3]*, a single copy insertion on chromosome II. The endogenous *C. elegans* plastin ortholog, *plst-1*, was intact. Control single copy insertion *rtSi28* did not express PLS3, but was inserted at the same location on II, confirming that insertion into this locus had no impact on *unc-25* locomotion in this assay. n≥30 animals per determination, combined from 3 independent trials. ANOVA *F*(7.37,7.45) = 20.09, *p<0.001* Mann-Whitney U-test: **p<0.01, S.E.M indicated. b) PLS3 overexpression is unable to rescue the unc phenotype in *unc-13* animals when assessing dispersal on food. Animals are reported as the percentage of animals that escaped the center ring after 1, 2 or 3 hours. Mean percent of animals escaped is calculated from three independent trials of at least 24 animals per genotype, per trial. Each trial is shown as a grey circle. **Figure S6.** Uncropped western blots. Panels a, d and e correspond to Fig. 5 panels a, b and c, respectively. Panels b and c correspond to supplementary figure 3. **Table S1.** Strains.

## Data Availability

New *C. elegans* strains generated here are available from the Hart laboratory and/or the CGC. Plasmids are available from the Hart laboratory. Data supporting all of the figures will be made available at a permanent DOI location via Brown University upon publication.

## References

[1] Fischer U, Liu Q, Dreyfuss G (1997). The SMN-SIP1 complex has an essential role in spliceosomal snRNP biogenesis. Cell.

[2] Liu Q, Fischer U, Wang F, Dreyfuss G (1997). The spinal muscular atrophy disease gene product, SMN, and its associated protein SIP1 are in a complex with spliceosomal snRNP proteins. Cell.

[3] Pellizzoni L, Yong J, Dreyfuss G (2002). Essential role for the SMN complex in the specificity of snRNP assembly. Science.

[4] Zou T, Yang X, Pan D, Huang J, Sahin M, Zhou J (2011). SMN deficiency reduces cellular ability to form stress granules, sensitizing cells to stress. Cell Mol Neurobiol.

[5] Hua Y, Zhou J (2004). Rpp20 interacts with SMN and is re-distributed into SMN granules in response to stress. Biochem Biophys Res Commun.

[6] Fallini C, Zhang HL, Su YH, Silani V, Singer RH, Rossoll W, Bassell GJ (2011). The survival of motor neuron (SMN) protein interacts with the mRNA-binding protein HuD and regulates localization of poly(A) mRNA in primary motor neuron axons. J Neurosci.

[7] Akten B, Kye MJ, Hao LT, Wertz MH, Singh S, Nie DY, Huang J, Merianda TT, Twiss JL, Beattie CE (2011). Interaction of survival of motor neuron (SMN) and HuD proteins with mRNA cpg15 rescues motor neuron axonal deficits. P Natl Acad Sci USA.

[8] Dimitriadi M, Derdowski A, Kalloo G, Maginnis MS, O'Hern P, Bliska B, Sorkac A, Nguyen KC, Cook SJ, Poulogiannis G (2016). Decreased function of survival motor neuron protein impairs endocytic pathways. Proc Natl Acad Sci U S A.

[9] Hosseinibarkooie S, Peters M, Torres-Benito L, Rastetter RH, Hupperich K, Hoffmann A, Mendoza-Ferreira N, Kaczmarek A, Janzen E, Milbradt J (2016). The power of human protective modifiers: PLS3 and CORO1C unravel impaired endocytosis in spinal muscular atrophy and rescue SMA phenotype. Am J Hum Genet.

[10] Riessland M, Kaczmarek A, Schneider S, Swoboda KJ, Lohr H, Bradler C, Grysko V, Dimitriadi M, Hosseinibarkooie S, Torres-Benito L (2017). Neurocalcin delta suppression protects against spinal muscular atrophy in humans and across species by restoring impaired endocytosis. Am J Hum Genet.

[11] O'Hern PJ, do Carmo GGI, Brecht J, Lopez Soto EJ, Simon J, Chapkis N, Lipscombe D, Kye MJ, Hart AC: Decreased microRNA levels lead to deleterious increases in neuronal M2 muscarinic receptors in Spinal Muscular Atrophy models. Elife. 2017;6:e20752. 10.7554/eLife.20752. 10.7554/eLife.20752 PMC5413352 28463115

[12] Oprea GE, Krober S, McWhorter ML, Rossoll W, Muller S, Krawczak M, Bassell GJ, Beattie CE, Wirth B (2008). Plastin 3 is a protective modifier of autosomal recessive spinal muscular atrophy. Science.

[13] Delanote V, Vandekerckhove J, Gettemans J (2005). Plastins: versatile modulators of actin organization in (patho) physiological cellular processes. Acta Pharmacol Sin.

[14] Engqvist-Goldstein AE, Drubin DG (2003). Actin assembly and endocytosis: from yeast to mammals. Annu Rev Cell Dev Biol.

[15] McGovern VL, Massoni-Laporte A, Wang X, Le TT, Le HT, Beattie CE, Rich MM, Burghes AH (2015). Plastin 3 expression does not modify spinal muscular atrophy severity in the 7 SMA mouse. PLoS One.

[16] Ackermann B, Krober S, Torres-Benito L, Borgmann A, Peters M, Barkooie SMH, Tejero R, Jakubik M, Schreml J, Milbradt J (2013). Plastin 3 ameliorates spinal muscular atrophy via delayed axon pruning and improves neuromuscular junction functionality. Hum Mol Genet.

[17] Alrafiah A, Karyka E, Coldicott I, Iremonger K, Lewis KE, Ning K, Azzouz M (2018). Plastin 3 promotes motor neuron axonal growth and extends survival in a mouse model of spinal muscular atrophy. Mol Ther Methods Clin Dev.

[18] Kaifer KA, Villalón E, Osman EY, Glascock JJ, Arnold LL, DDW C, Lorson CL (2017). Plastin-3 extends survival and reduces severity in mouse models of spinal muscular atrophy. JCI Insight.

[19] Janzen E, Mendoza-Ferreira N, Hosseinibarkooie S, Schneider S, Hupperich K, Tschanz T, Grysko V, Riessland M, Hammerschmidt M, Rigo F, Bennett CF, Kye MJ, Torres-Benito L, Wirth B (2018). CHP1 reduction ameliorates spinal muscular atrophy pathology by restoring calcineurin activity and endocytosis. Brain.

[20] Janzen E, Wolff L, Mendoza-Ferreira N, Hupperich K, DelleVedove A, Hosseinibarkooie S, Kye MJ, Wirth B (2019). PLS3 overexpression delays ataxia in *Chp1* mutant mice. Front Neurosci.

[21] Cooper TA, Wan L, Dreyfuss G (2009). RNA and disease. Cell.

[22] Khalil B, Morderer D, Price PL, Liu F, Rossoll W (2018). mRNP assembly, axonal transport, and local translation in neurodegenerative diseases. Brain Res.

[23] Liu-Yesucevitz L, Bassell GJ, Gitler AD, Hart AC, Klann E, Richter JD, Warren ST, Wolozin B (2011). Local RNA translation at the synapse and in disease. J Neurosci.

[24] Nizzardo M, Taiana M, Rizzo F, Aguila Benitez J, Nijssen J, Allodi I, Melzi V, Bresolin N, Comi GP, Hedlund E, Corti S. Synaptotagmin 13 is neuroprotective across motor neuron diseases. Acta Neuropathol. 2020. 10.1007/s00401-020-02133-x PMC7181443 32065260

[25] Hao le, T., H. R. Fuller, T. Lam le, T. T. Le, A. H. Burghes and G. E. Morris: Absence of gemin5 from SMN complexes in nuclear Cajal bodies. BMC Cell Biol 2007. 8: 28. 10.1186/1471-2121-8-28 PMC1939999 17640370

[26] Cacciottolo R, Ciantar J, Lanfranco M, Borg RM, Vassallo N, Bordonne R, Cauchi RJ (2019). SMN complex member Gemin3 self-interacts and has a functional relationship with ALS-linked proteins TDP-43, FUS and Sod1. Sci Rep.

[27] Gertz B, Wong M, Martin LJ (2012). Nuclear localization of human SOD1 and mutant SOD1-specific disruption of survival motor neuron protein complex in transgenic amyotrophic lateral sclerosis mice. J Neuropathol Exp Neuro.

[28] Kariya S, Re DB, Jacquier A, Nelson K, Przedborski S, Monani UR (2012). Mutant superoxide dismutase 1 (SOD1), a cause of amyotrophic lateral sclerosis, disrupts the recruitment of SMN, the spinal muscular atrophy protein to nuclear Cajal bodies. Hum Mol Genet.

[29] Yanagi KS, Wu Z, Amaya J, Chapkis N, Duffy AM, Hajdarovic KH, Held A, Mathur AD, Russo K, Ryan VH, Steinert BL, Whitt JP, Fallon JR, Fawzi NL, Lipscombe D, Reenan RA, Wharton KA, Hart AC (2019). Meta-analysis of genetic modifiers reveals candidate dysregulated pathways in amyotrophic lateral sclerosis. Neuroscience.

[30] Wang J, Farr GW, Hall DH, Li F, Furtak K, Dreier L, Horwich AL: An ALS-linked mutant SOD1 produces a locomotor defect associated with aggregation and synaptic dysfunction when expressed in neurons of *Caenorhabditis elegans*. PLoS Genet. 2009;5(1):e1000350. 10.1371/journal.pgen.1000350. 10.1371/journal.pgen.1000350 PMC2621352 19165329

[31] Briese M, Esmaeili B, Fraboulet S, Burt EC, Christodoulou S, Towers PR, Davies KE, Sattelle DB (2009). Deletion of smn-1, the Caenorhabditis elegans ortholog of the spinal muscular atrophy gene, results in locomotor dysfunction and reduced lifespan. Hum Mol Genet.

[32] Miguel-Aliaga I, Culetto E, Walker DS, Baylis HA, Sattelle DB, Davies KE (1999). The Caenorhabditis elegans orthologue of the human gene responsible for spinal muscular atrophy is a maternal product critical for germline maturation and embryonic viability. Hum Mol Genet.

[33] Sleigh JN, Buckingham SD, Cuppen E, Viswanathan M, Westlund BM, Sattelle DB (2010). A novel point mutation in the Caenorhabditis elegans smn-1 gene provides a useful model for investigating Spinal Muscular Atrophy. Neuromuscul Disord.

[34] Sun Y, Grimmler M, Schwarzer V, Schoenen F, Fischer U, Wirth B (2005). Molecular and functional analysis of intragenic SMN1 mutations in patients with spinal muscular atrophy. Hum Mutat.

[35] Sleigh JN, Buckingham SD, Esmaeili B, Viswanathan M, Cuppen E, Westlund BM, Sattelle DB (2011). A novel Caenorhabditis elegans allele, smn-1(cb131), mimicking a mild form of spinal muscular atrophy, provides a convenient drug screening platform highlighting new and pre-approved compounds. Hum Mol Genet.

[36] Liewald JF, Brauner M, Stephens GJ, Bouhours M, Schultheis C, Zhen M, Gottschalk A (2008). Optogenetic analysis of synaptic function. Nat Methods.

[37] Liu Q, Hollopeter G, Jorgensen EM (2009). Graded synaptic transmission at the Caenorhabditis elegans neuromuscular junction. P Natl Acad Sci USA.

[38] Hao LT, Wolman M, Granato M, Beattie CE (2012). Survival motor neuron affects plastin 3 protein levels leading to motor defects. J Neurosci.

[39] Dimitriadi M, Sleigh JN, Walker A, Chang HC, Sen A, Kalloo G, Harris J, Barsby T, Walsh MB, Satterlee JS, Li C, Van Vactor D, Artavanis-Tsakonas S, Hart AC. Conserved genes act as modifiers of invertebrate SMN loss of function defects. PLoS genetics. 2010;6(10):e1001172. 10.1371/journal.pgen.1001172. 10.1371/journal.pgen.1001172 PMC2965752 21124729

[40] Sen A, Dimlich DN, Guruharsha KG, Kankel MW, Hori K, Yokokura T, Brachat S, Richardson D, Loureiro J, Sivasankaran R (2013). Genetic circuitry of survival motor neuron, the gene underlying spinal muscular atrophy. P Natl Acad Sci USA.

[41] Hsu DR, Chuang PT, Meyer BJ (1995). DPY-30, a nuclear protein essential early in embryogenesis for Caenorhabditis elegans dosage compensation. Development.

[42] Dreyfuss G, Kim VN, Kataoka N (2002). Messenger-RNA-binding proteins and the messages they carry. Nat Rev Mol Cell Bio.

[43] Dearruda MV, Watson S, Lin CS, Leavitt J, Matsudaira P (1990). Fimbrin is a homolog of the cytoplasmic phosphoprotein plastin and has domains homologous with calmodulin and actin gelation proteins. J Cell Biol.

[44] Sandrock TM, Brower SM, Toenjes KA, Adams AEM (1999). Suppressor analysis of fimbrin (Sac6p) overexpression in yeast. Genetics.

[45] Guruharsha KG, Rual JF, Zhai B, Mintseris J, Vaidya P, Vaidya N, Beekman C, Wong C, Rhee DY, Cenaj O, McKillip E, Shah S, Stapleton M, Wan KH, Yu C, Parsa B, Carlson JW, Chen X, Kapadia B, Vijayraghavan K, Gygi SP, Celniker SE, Obar RA, Artavanis-Tsakonas S (2011). A protein complex network of Drosophila melanogaster. Cell.

[46] Yochem J, Bell LR, Herman RK (2004). The identities of sym-2, sym-3 and sym-4, three genes that are synthetically lethal with mec-8 in Caenorhabditis elegans. Genetics.

[47] Dreyfuss G, Matunis MJ, Pinolroma S, Burd CG (1993). Hnrnp proteins and the biogenesis of messenger-Rna. Annu Rev Biochem.

[48] Liu Y, Gervasi C, Szaro BG (2008). A crucial role for hnRNP K in axon development in Xenopus laevis. Development.

[49] Pagliardini S, Giavazzi A, Setola V, Lizier C, Di Luca M, DeBiasi S, Battaglia G (2000). Subcellular localization and axonal transport of the survival motor neuron (SMN) protein in the developing rat spinal cord. Hum Mol Genet.

[50] Rossoll W, Kroning AK, Ohndorf UM, Steegborn C, Jablonka S, Sendtner M (2002). Specific interaction of Smn, the spinal muscular atrophy determining gene product, with hnRNP-R and gry-rbp/hnRNP-Q: a role for Smn in RNA processing in motor axons?. Hum Mol Genet.

[51] Gupta SK, Kosti I, Plaut G, Pivko A, Tkacz ID, Cohen-Chalamish S, Biswas DK, Wachtel C, Ben-Asher HW, Carmi S (2013). The hnRNP F/H homologue of Trypanosoma brucei is differentially expressed in the two life cycle stages of the parasite and regulates splicing and mRNA stability. Nucleic Acids Res.

[52] Wang E, Aslanzadeh V, Papa F, Zhu H, de la Grange P, Cambi F. Global profiling of alternative splicing events and gene expression regulated byhnRNPH/F. PLoS One. 2012;7(12):e51266. 10.1371/journal.pone.0051266. 10.1371/journal.pone.0051266 PMC3524136 23284676

[53] Fitzgerald K, Greenwald I (1995). Interchangeability of Caenorhabditis elegans DSL proteins and intrinsic signalling activity of their extracellular domains in vivo. Development.

[54] Grant B, Greenwald I (1997). Structure, function, and expression of SEL-1, a negative regulator of LIN-12 and GLP-1 in C. elegans. Development.

[55] Fares H, Greenwald I (2001). Genetic analysis of endocytosis in Caenorhabditis elegans: coelomocyte uptake defective mutants. Genetics.

[56] Nishimura AL, Mitne-Neto M, Silva HCA, Richieri-Costa A, Middleton S, Cascio D, Kok F, Oliveira JRM, Gillingwater T, Webb J (2004). A mutation in the vesicle-trafficking protein VAPB causes late-onset spinal muscular atrophy and amyotrophic lateral sclerosis. Am J Hum Genet.

[57] Bastow E, Peswani AR, Tarrant D, Pentland DR, Chen X, Morgan A, Staniforth G, Tullet JM, Rowe ML, Howard MF (2016). New links between SOD1 and metabolic dysfunction from a yeast model of amyotrophic lateral sclerosis. J Cell Sci.

[58] Jin YS, Jorgensen E, Hartwieg E, Horvitz HR (1999). The Caenorhabditis elegans gene unc-25 encodes glutamic acid decarboxylase and is required for synaptic transmission but not synaptic development. J Neurosci.

[59] Yu A, Shibata Y, Shah B, Calamini B, Lo DC, Morimoto RI (2014). Protein aggregation can inhibit clathrin-mediated endocytosis by chaperone competition. Proc Natl Acad Sci U S A.

[60] Urwin H, Authier A, Nielsen JE, Metcalf D, Powell C, Froud K, Malcolm DS, Holm I, Johannsen P, Brown J (2010). Disruption of endocytic trafficking in frontotemporal dementia with CHMP2B mutations. Hum Mol Genet.

[61] Brenner S (1974). Genetics of Caenorhabditis-elegans. Genetics.

[62] Calixto A, Chelur D, Topalidou I, Chen XY, Chalfie M (2010). Enhanced neuronal RNAi in C. elegans using SID-1. Nat Methods.

[63] Frokjaer-Jensen C, Davis MW, Hopkins CE, Newman BJ, Thummel JM, Olesen SP, Grunnet M, Jorgensen EM (2008). Single-copy insertion of transgenes in Caenorhabditis elegans. Nat Genet.

[64] Fukushige T, Hawkins MG, McGhee JD (1998). The GATA-factor elt-2 is essential for formation of the Caenorhabditis elegans intestine. Dev Biol.

[65] Krajacic P, Shen XN, Purohit PK, Arratia P, Lamitina T (2012). Biomechanical profiling of Caenorhabditis elegans motility. Genetics.

[66] Kamath RS, Fraser AG, Dong Y, Poulin G, Durbin R, Gotta M, Kanapin A, Le Bot N, Moreno S, Sohrmann M (2003). Systematic functional analysis of the Caenorhabditis elegans genome using RNAi. Nature.

[67] Mahoney TR, Luo S, Nonet ML (2006). Analysis of synaptic transmission in Caenorhabditis elegans using an aldicarb-sensitivity assay. Nat Protoc.

[68] Wichterle H, Lieberam I, Porter JA, Jessell TM (2002). Directed differentiation of embryonic stem cells into motor neurons. Cell.

