# Supplementary material for: Genetic modifiers ameliorate endocytic and neuromuscular defects in a model of spinal muscular atrophy
Source: BMC Biol. 2020 Sep 16;18:127. doi: 10.1186/s12915-020-00845-w (PMC7495824; doi:10.1186/s12915-020-00845-w)

Additional file 1: Supplementary Figure 1

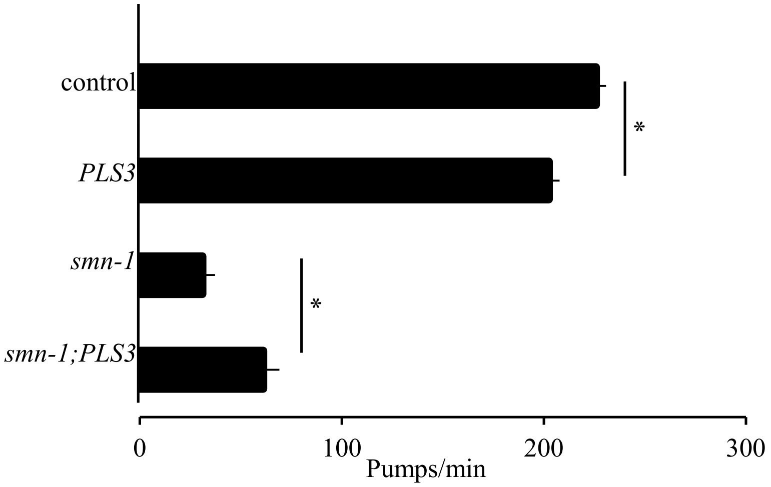

Additional file 1: Supplementary Figure 2

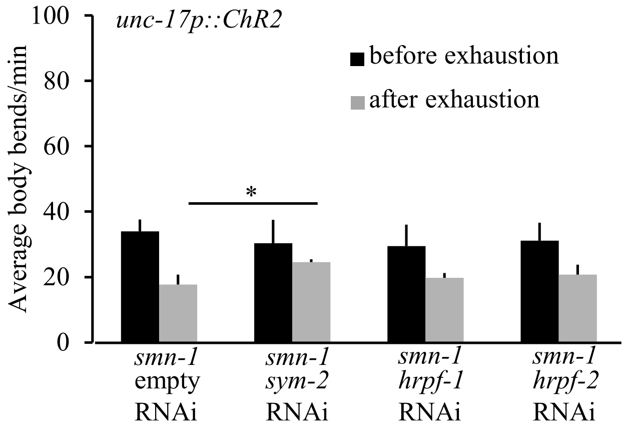

Additional file 1: Supplementary Figure 3

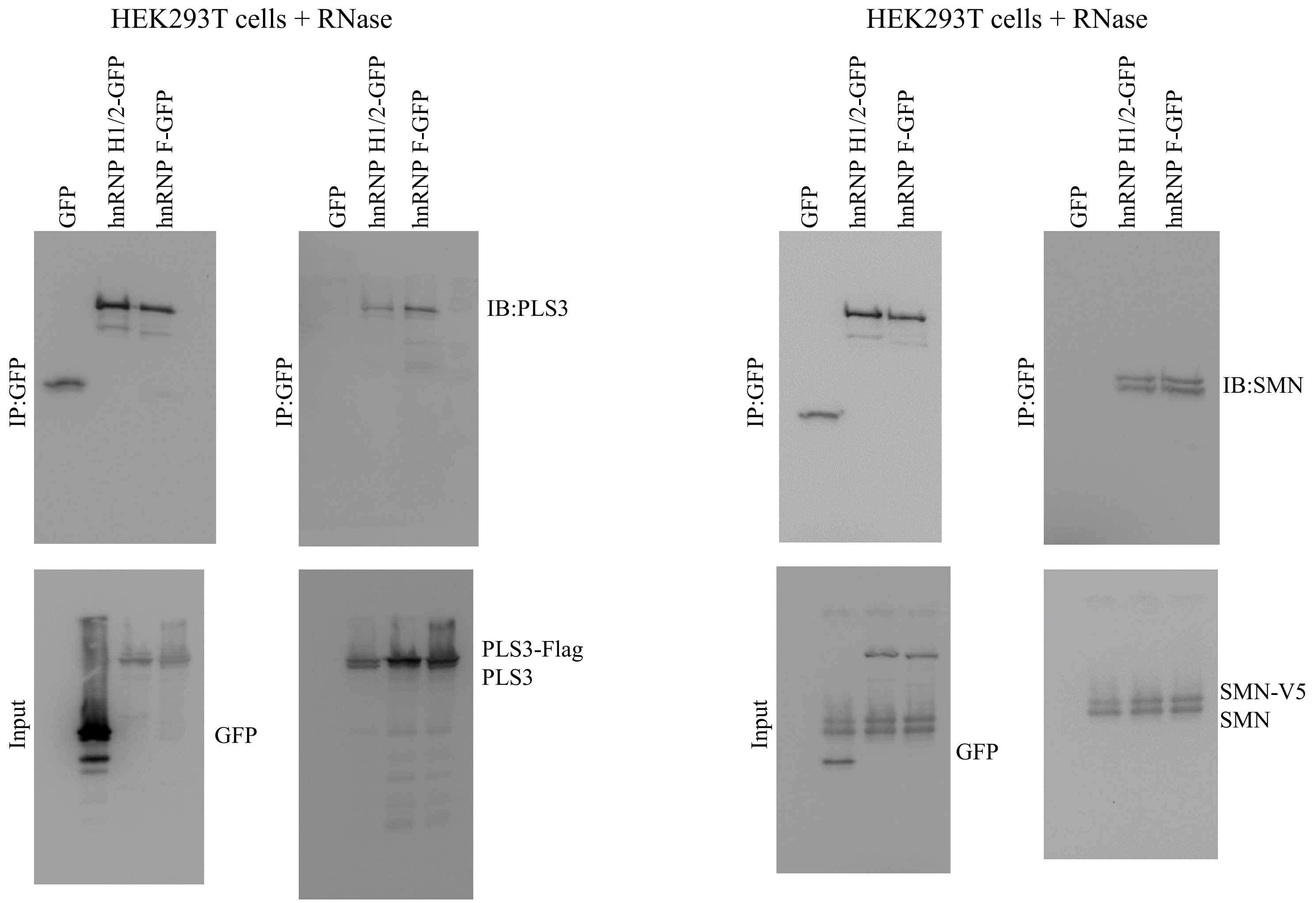

Additional file 1: Supplementary Figure 4

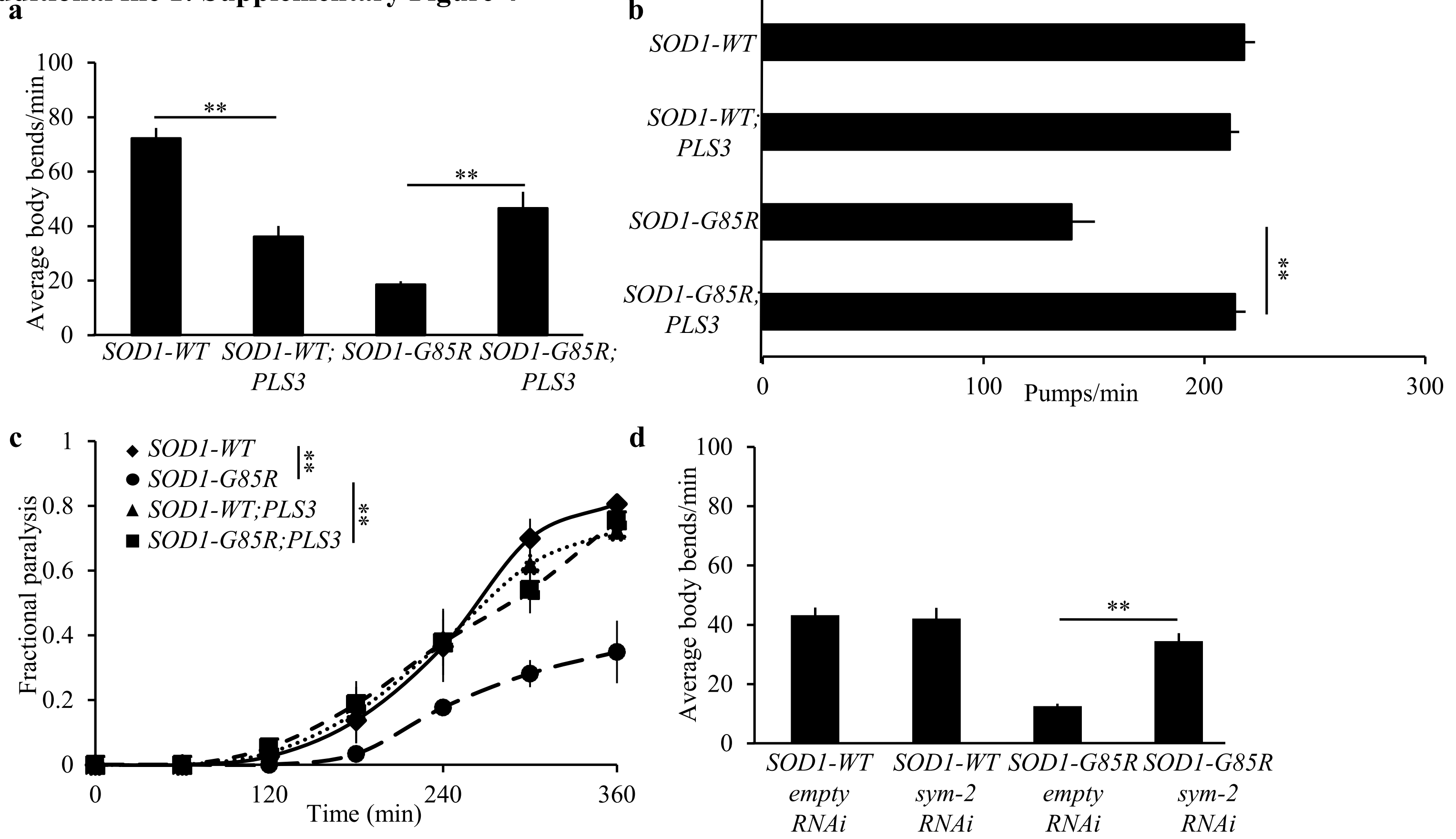

Additional file 1: Supplementary Figure 5

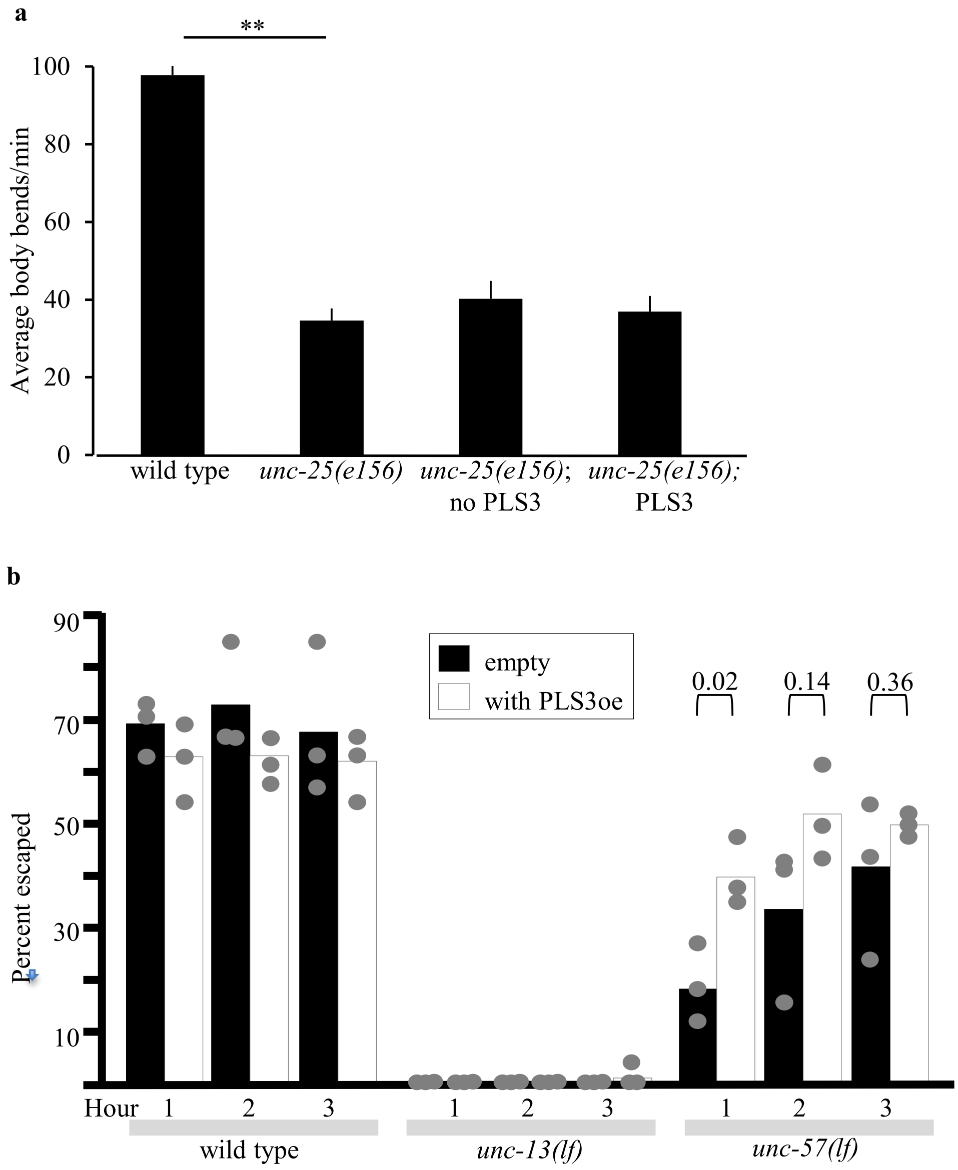

Additional file 1:
Supplementary Figure 6

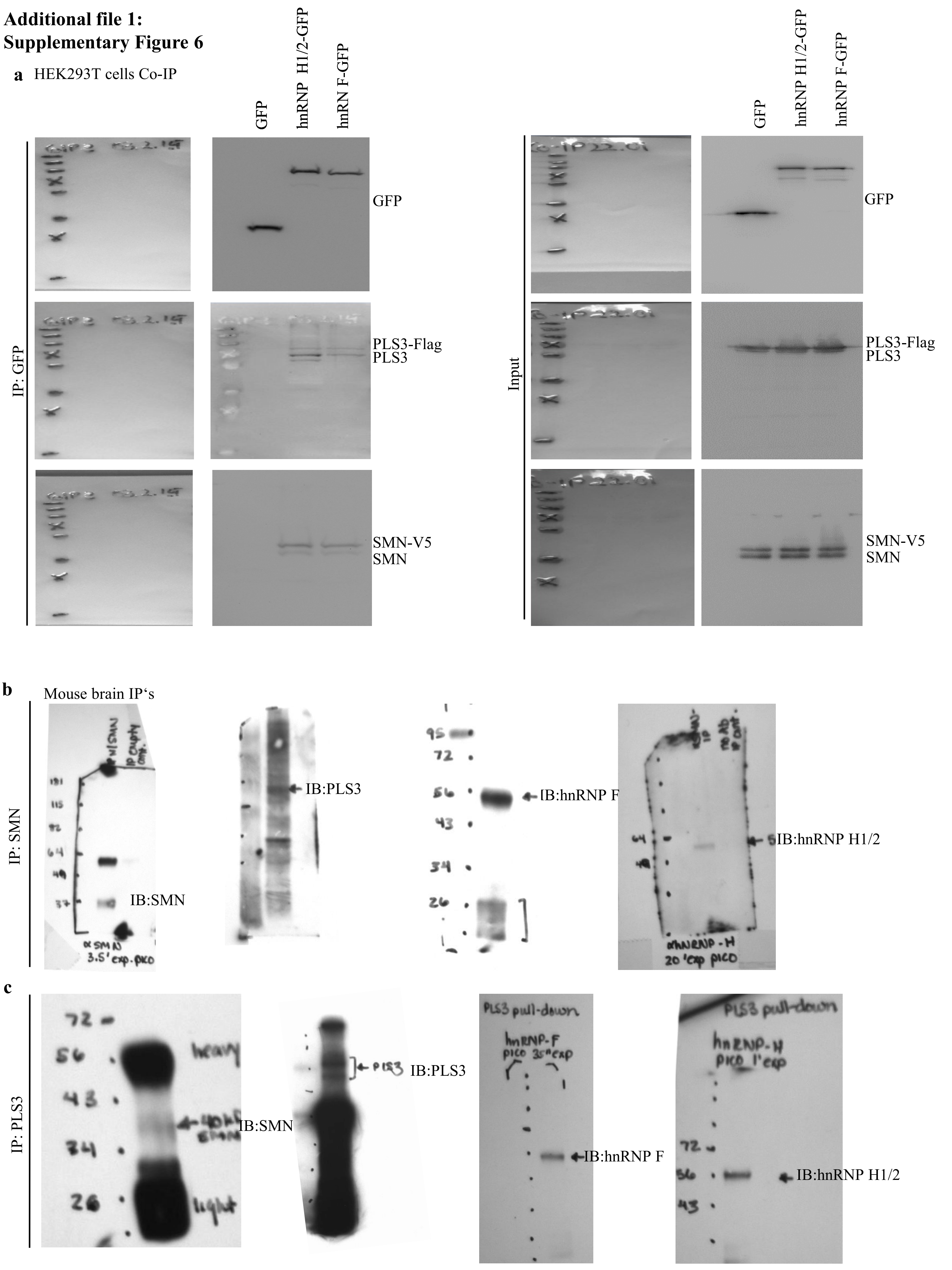

d HEK293T cells Co-IP with RNase

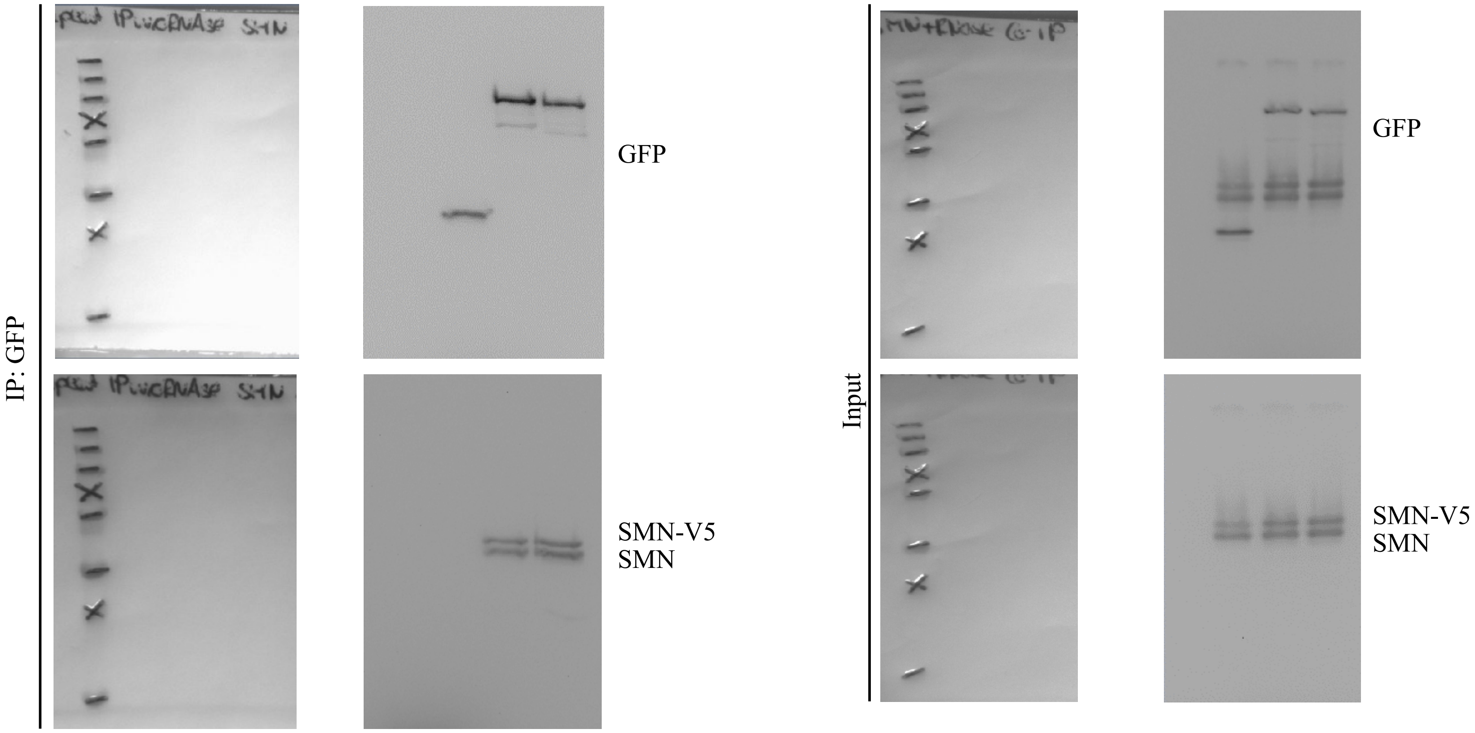

e HEK293T cells Co-IP with RNase

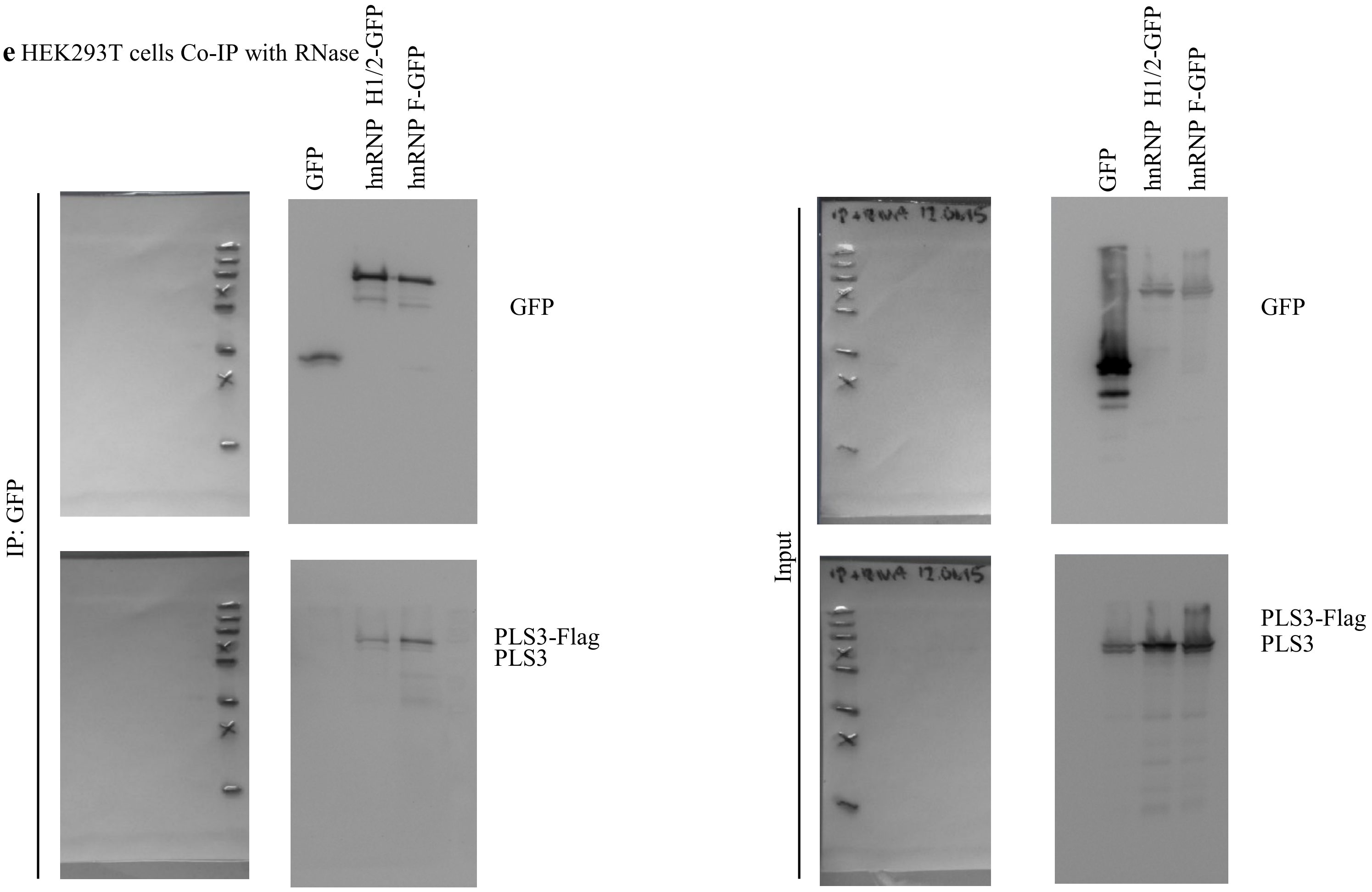

Supplement: Supplementary file 1 — Additional file 1: Figure S1. PLS3 overexpression suppressed pharyngeal pumping defects in smn-1(ok355) animals. Homozygous null smn-1(ok355) animals lacking smn-1 survive through early larval stages due to maternal loading of SMN-1 proteins and mRNA by heterozygous smn-1(ok355)/hT2 mothers. hT2 carries a functional copy of endogenous smn-1, overexpression of human PLS3 slightly lowered pumping rates in control smn-1(+) animals, but increased pumping rates in homozygous smn-1(ok355) animals (compared to control smn-1(ok355)). To control for genetic background all animals were derived from mothers heterozygous for hT2. To control for transgene insertion position, control and smn-1(ok355) animals carried rtSi28 [dpy-30p::empty]. PLS3 is overexpressed from rtSi27 [dpy-30p::PLS3], a single copy insertion on chromosome II. n≥30 animals per determination, combined from 3 independent trials that the scorer was blinded to the genotype of animals. ANOVA F(9.9,13.1) = 14.23, p<0.001; post-hoc Mann-Whitney U-test *p<0.05, S.E. indicated. Figure S2. sym-2 knockdown using RNAi suppressed the smn-1 locomotion defect. Exhaustion of cholinergic motor neurons using ChR2 slowed locomotion; smn-1(cb131) animals with decreased SMN-1 function had aberrantly low locomotion rates post-exhaustion. RNAi knockdown of hnRNP F/H ortholog sym-2 ameliorated this defect. empty (RNAi) used as a control as the bacterial strain used for RNAi can alter locomotion rates. n≥30 animals per determination, combined from 3 independent trials. Student’s t-test *p<0.05 S.E.M. indicated. Figure S3. Association of SMN and PLS3 with hnRNPF or hnRNPH1/2, is not dependent on RNA. Pretreatment with RNase had no impact on co-immunoprecipitation of PLS3 or SMN from HEK293T cells using GFP tagged hnRNP F or hnRNP H1/2. SMN was tagged with V5; PLS3 was tagged with Flag. Conditions and procedures as described in Fig. 4a. Figure S4.PLS3 and sym-2 suppress behavioral defects in specific C. elegans models of neurodegenera [file 12915_2020_845_MOESM1_ESM.zip › Additional files.pdf]
