# Supplementary material for: Genetic modifiers ameliorate endocytic and neuromuscular defects in a model of spinal muscular atrophy
Source: BMC Biol. 2020 Sep 16;18:127. doi: 10.1186/s12915-020-00845-w (PMC7495824; doi:10.1186/s12915-020-00845-w)
Supplement: Supplementary file 1 — Additional file 1: Figure S1. PLS3 overexpression suppressed pharyngeal pumping defects in smn-1(ok355) animals. Homozygous null smn-1(ok355) animals lacking smn-1 survive through early larval stages due to maternal loading of SMN-1 proteins and mRNA by heterozygous smn-1(ok355)/hT2 mothers. hT2 carries a functional copy of endogenous smn-1, overexpression of human PLS3 slightly lowered pumping rates in control smn-1(+) animals, but increased pumping rates in homozygous smn-1(ok355) animals (compared to control smn-1(ok355)). To control for genetic background all animals were derived from mothers heterozygous for hT2. To control for transgene insertion position, control and smn-1(ok355) animals carried rtSi28 [dpy-30p::empty]. PLS3 is overexpressed from rtSi27 [dpy-30p::PLS3], a single copy insertion on chromosome II. n≥30 animals per determination, combined from 3 independent trials that the scorer was blinded to the genotype of animals. ANOVA F(9.9,13.1) = 14.23, p<0.001; post-hoc Mann-Whitney U-test *p<0.05, S.E. indicated. Figure S2. sym-2 knockdown using RNAi suppressed the smn-1 locomotion defect. Exhaustion of cholinergic motor neurons using ChR2 slowed locomotion; smn-1(cb131) animals with decreased SMN-1 function had aberrantly low locomotion rates post-exhaustion. RNAi knockdown of hnRNP F/H ortholog sym-2 ameliorated this defect. empty (RNAi) used as a control as the bacterial strain used for RNAi can alter locomotion rates. n≥30 animals per determination, combined from 3 independent trials. Student’s t-test *p<0.05 S.E.M. indicated. Figure S3. Association of SMN and PLS3 with hnRNPF or hnRNPH1/2, is not dependent on RNA. Pretreatment with RNase had no impact on co-immunoprecipitation of PLS3 or SMN from HEK293T cells using GFP tagged hnRNP F or hnRNP H1/2. SMN was tagged with V5; PLS3 was tagged with Flag. Conditions and procedures as described in Fig. 4a. Figure S4.PLS3 and sym-2 suppress behavioral defects in specific C. elegans models of neurodegenera [file 12915_2020_845_MOESM1_ESM.zip › Table S1.docx]

| Strain name | *Genotype* |
| --- | --- |
| EG5425 | *oxIs364[unc-17p::channelrhodopsin2::mCherry + lin-15(+) + Litmus]X* |
| HA2163 | *smn-1(cb131)I;oxIs364X* |
| LM99 | *smn-1(ok355)I/hT2(I;III)* |
| HA1981 | *+/hT2(I;III)* |
| HA2245 | *smn-1(cb131)I;rtSi27[dpy-30p::PLS3::unc-54UTR, unc-119(+)](IV);oxIs364X* |
| HA2246 | *smn-1(cb131)I; rtSi28[dpy-30p::unc-54UTR](IV);oxIs364X* |
| HA2243 | *smn-1(cb131)I;rtIs59[dpy-30p::PLS3::unc-54UTR];oxIs364X* |
| HA2244 | *smn-1(cb131)I;rtEx850[dpy-30p::plst-1::unc-54UTR];oxIs364X* |
| SP2230 | *sym-2(mn617)II* |
| HA2686 | *sym-2(mn617)II;oxIs364X* |
| HA2687 | *smn-1(cb131)I;sym-2(mn617)II;oxIs364X* |
| HA2682 | *smn-1(cb131)I;rtEx851[myo-3p::PLS3::unc-54UTR, unc-119(+)];oxIs364X* |
| HA2683 | *smn-1(cb131); rtEx853[myo-3p::unc-54UTR];oxIs364X* |
| HA2684 | *smn-1(cb131)I ; rtEx852[unc119p::PLS3::unc-54UTR, unc-119(+)];oxIs364X* |
| HA2685 | *smn-1(cb131)I ; rtEx854[unc119p::unc-54UTR];oxIs364X* |
| HA2627 | *+/hT2(I;III);rtSi27[dpy-30p::PLS3::unc-54UTR, unc-119(+)](IV)* |
| HA2629 | *+/hT2(I;III);rtSi28[dpy-30p::unc-54UTR](IV)* |
| HA2628 | *smn-1(ok355)/hT2(I;III); rtSi28[dpy-30p::unc-54UTR](IV)* |
| HA2626 | *smn-1(ok355)/hT2(I;III); rtSi27[dpy-30p::PLS3::unc-54UTR, unc-119(+)](IV)* |
| HA2688 | *sym-2(mn617)II; rtSi27[dpy-30p::PLS3::unc-54UTR, unc-119(+)](IV)* |
| HA2689 | *sym-2(mn617)II; rtSi28[dpy-30p::unc-54UTR](IV)* |
| AM82 | *rmEx95[pF25B3.3::Q19::CFP]* |
| AM81 | *rmEx164 [pF25B3.3p::Q67::CFP]* |
| CB156 | *unc-25(e156)III.* |
| LC108 | *uls69[pCFJ90(myo-2p::mCherry), unc 119p::sid-1]* |
| SOD1-WT | *Psnb1::human SOD1-YFP* |
| SOD1-G85R | *Psnb1::humanSOD1-G85R-YFP* |
| HA2690 | *Psnb1::human SOD1-YFP;rtSi27[dpy-30p::PLS3::unc-54UTR, unc-119(+)](IV)* |
| HA2691 | *Psnb1::human SOD1-YFP; rtSi28[dpy-30p::unc-54UTR](IV)* |
| HA2692 | *Psnb1::humanSOD1-G85R-YFP;rtSi27[dpy-30p::PLS3::unc-54UTR, unc-119(+)](IV)* |
| HA2693 | *Psnb1::humanSOD1-G85R-YFP;rtSi28[dpy-30p::unc-54UTR](IV)* |
| HA2694 | *rmEx95[pF25B3.3::Q19::CFP];rtSi27[dpy-30p::PLS3::unc-54UTR, unc-119(+)](IV)* |
| HA2695 | *rmEx95[pF25B3.3::Q19::CFP];rtSi28[dpy-30p::unc-54UTR](IV)* |
| HA2696 | *rmEx164 [pF25B3.3p::Q67::CFP];rtSi27[dpy-30p::PLS3::unc-54UTR, unc-119(+)](IV)* |
| HA2697 | *rmEx164 [pF25B3.3p::Q67::CFP];rtSi28[dpy-30p::unc-54UTR](IV)* |
| GS1912 | *arIs37[myo-3p::ssGFP+dpy-20(+)]Ildot-20(e1282)IV* |
| HA3382 | *arIs37[myo-3p::ssGFP+dpy-20(+)]Ildot-20(e1282)IV;rtEx906[unc-122p::smn-1RNAi+coel::RFP+ unc-122p::empty }* |
| HA3383 | *arIs37[myo-3p::ssGFP+dpy-20(+)]Ildot-20(e1282)IV;rtEx907[unc-122p::empty+coel::RFP}* |
| HA3384 | *arIs37[myo-3p::ssGFP+dpy-20(+)]Ildot-20(e1282)IV;rtEx908[unc-122p::smn-1RNAi+ unc-122p::PLS3+coel::RFP}* |
| HA3385 | *arIs37[myo-3p::ssGFP+dpy-20(+)]Ildot-20(e1282)IV;rtEx909[unc-122p::PLS3+ unc-122p::empty +coel::RFP}* |
| HA3386 | *arIs37[myo-3p::ssGFP+dpy-20(+)]Ildot-20(e1282)IV;rtEx910[unc-122p::smn-1RNAi+ unc-122p::sym-2RNAi +coel::RFP}* |
| HA3387 | *arIs37[myo-3p::ssGFP+dpy-20(+)]Ildot-20(e1282)IV;rtEx911[unc-122p::sym-2RNAi+unc-122p::empty +coel::RFP}* |
